# A discrete time simulation model for kidney allocation in Germany

**DOI:** 10.3389/ti.2026.16189

**Published:** 2026-05-28

**Authors:** Nassim Kakavand, Maarten Coemans, Hans C. de Ferrante, Benedikt Kolbrink, Malte Ziemann, David I. Radke, Matthias Lindner, Maarten Naesens, Roland Schmitt, Kevin Schulte, Friedrich A. von Samson-Himmelstjerna

**Affiliations:** 1 Department of Anesthesiology and Intensive Care Medicine, University Medical Center Schleswig-Holstein, Christian-Albrechts-University, Kiel, Germany; 2 Department of Microbiology, Immunology and Transplantation, KU Leuven, Leuven, Belgium; 3 Department of Mathematics and Computer Science, Eindhoven University of Technology, Eindhoven, Netherlands; 4 Eurotransplant International Foundation, Leiden, Netherlands; 5 Department of Nephrology and Hypertension, University Medical Center Schleswig-Holstein, Christian-Albrechts-University, Kiel, Germany; 6 Institute for Transfusion Medicine, University Medical Center Schleswig-Holstein, Universität zu Lübeck, Lübeck, Germany

**Keywords:** discrete time simulation, Eurotransplant, Germany, kidney allocation, simulated allocation models

## Abstract

The ongoing shortage of kidney donations increases pressure to optimize utility and equity in kidney allocation; simulation models allow evaluation of policy changes before implementation. Using data from the German national transplant registry (19,517 kidney transplants), human leukocyte antigen (HLA) information from the German bone marrow donor registry, and the Eurotransplant database, we developed a discrete time simulation model that reconstructs the current German kidney allocation process. To validate the model, we simulated the period from 2006 to 2017 and compared simulated and historical outputs. Waiting list size, numbers of removals and transplants, age distributions, HLA mismatch counts, and predicted long-term survival were highly similar to historic data, indicating solid calibration. As an exemplary application for the allocation model, we explored the effects of omitting the European Senior Program (ESP) from Eurotransplant kidney allocation. This alleviated disparities in waiting time for younger adults and slightly improved overall transplant survival rates, but it worsened access to transplantation for older patients. In conclusion, this discrete time simulation model provides a tool for assessing policy trade-offs on a variety of outcomes before clinical implementation. Further work is needed to generalize the model to the full Eurotransplant area.

## Graphical Abstract

**Figure:**
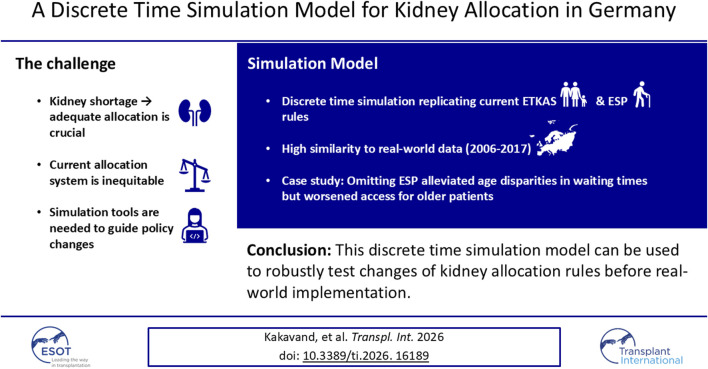

## Introduction

Patients with kidney failure face long waiting times for deceased donor kidneys. In Germany, waiting times in the Eurotransplant (ET) Kidney Allocation System (ETKAS) have increased since 2006 [1], currently reaching a median of around 9 years [2]. Despite kidney transplantation being the most effective treatment in terms of patient mortality and quality of life [3], the persistent scarcity of organs poses a major challenge for allocation.

The ET allocation program for deceased donor kidneys consists primarily of two distinct sub-programs: the ETKAS and the European Senior Program (ESP). In ETKAS, kidneys are allocated between donors and recipients younger than 65 years. Allocation is based on a scoring system, according to the following attributes: pediatric status, medical urgency, mismatch probability, human leukocyte antigen (HLA)-A, -B, -DR mismatches, waiting time, distance between donor- and transplant center, and national kidney exchange balance [4]. In case of imminent lack of access for dialysis a high urgency status can be requested. Pediatric patients till the age of 18 receive bonus points [4]. Organs from donors aged 65 or older are primarily allocated to recipients aged 65 years or older through the ESP. It aims to minimize cold ischemia time by prioritizing local and regional allocation. Ranking is based on urgency and waiting time, without considering HLA mismatches (except for unacceptable antigens) [4].

Within the ET network—one of the world’s largest organ exchange organizations [5]—balancing equity and utility remains of utmost importance [1]. Among other challenges, the rigid age thresholds for the ESP and pediatric bonus in Germany have led to age-related disparities in waiting times [1, 6]. Additionally, various proposals have been made to modify the HLA mismatching policy [7–12].

The current allocation algorithm was largely shaped by simulations conducted by Wujciak and Opelz in 1993 [13, 14]. An article published by ET in 1998 stated that “[…] any introduction of a change must be preceded by a computer simulation study […]” [15]. Since then, however, simulation efforts and changes to the allocation system within the ET area have been rare. With the exception of the model of Niemann et al., which focused exclusively on the feasibility of epitope matching within ETKAS [10], existing models fail to account for the evolving dynamics of transplant activity and waiting lists [16], or are tailored to targeted regional allocation programs [17]. In other regions, simulation models have been used more frequently and have facilitated changes to the allocation system. Examples can be found in France [18] and particularly in the United States with the “Kidney-pancreas simulated allocation model” (KPSAM) [19] and its predecessor the United Network for Organ Sharing Kidney Allocation Model (UKAM) [20]. All these models are tailored to specific regional policies and cannot easily be transferred to other allocation systems [16, 17].

The unavailability of simulation models tailored to the ET region has impeded changes to the allocation rules.

While a simulation model for kidney allocation within the ET area is currently under development [21], different modelling approaches help ensure that conclusions about allocation strategies are robust and not overly dependent on a single method or dataset. Our study addresses this gap by developing an independent, dynamic simulation model for ET-based kidney transplant allocation. The model is calibrated using German registry data and offers an adaptable tool for evaluating allocation strategies.

## Materials and methods

### Data

The model was developed using several complementary data sources. The German national transplant registry served as the primary source [22]. Data derived from the registry were cross-validated against annual reports published by ET and the German Organ Procurement Organization (Deutsche Stiftung Organspende, DSO) [23, 24]. Data on HLA allele and haplotype frequencies were derived from published sources based on data from the German bone marrow donor registry (DKMS) [25, 26]. The kidney offer acceptance behavior of transplant centers was modeled on ET data.

The German national transplant registry [22] is an anonymized, retrospective dataset on more than 52,000 solid organ transplantations performed in Germany from January 1, 2006, to January 1, 2017. After this, the registry has been maintained prospectively; however, the quality of the reported data completeness has concomitantly declined significantly [1, 27]. Given these limitations, we chose to use only the retrospective portion of the dataset. The registry includes information sourced from transplant centers, ET, and the DSO.

Patients transplanted with other organs than kidneys were excluded (6%, n = 1.688). For the waiting list, all patients co-listed on the pancreas waiting or liver waiting list were also excluded from the analysis (8%, n = 3.425). *En-bloc* kidney transplantations–approximately 1% of annual kidney transplants–were counted as a single transplanted kidney.

### Discrete time simulation

We modeled allocation using a discrete time simulation, where time advances in fixed increments rather than in response to events (as opposed to discrete event simulation).

Step sizes of 1/365, 1/36, 1/12 of a year, and 1 year were explored. No single increment size optimized all outcomes simultaneously. Smaller step sizes increased computational time, while larger ones reduced temporal precision. A step size of 1/36 of a year (≈10 days) provided a robust overall balance. The qualitative behavior of the model remained consistent across all step sizes, indicating that the simulation is not very sensitive to this choice.

**FIGURE 1:**
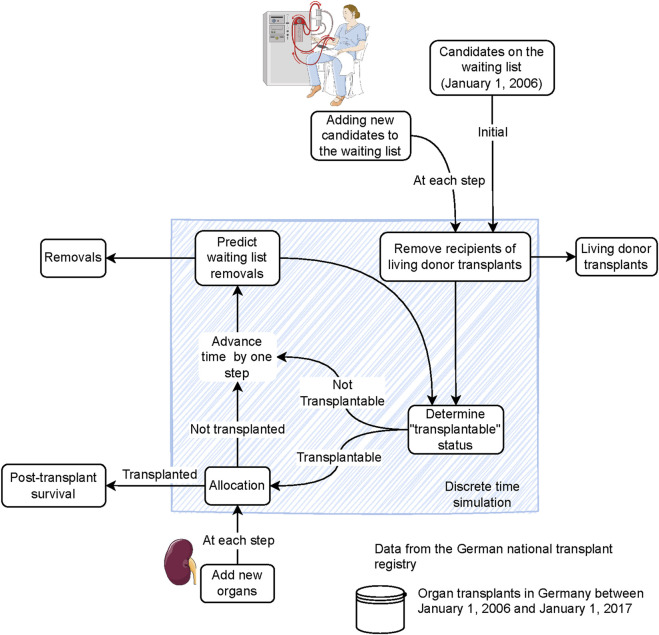
Simplified overview of the discrete time simulation model. Initially, newly added candidates are evaluated for the possibility of living donor transplantation. Next, each candidate’s transplantable status is determined. If deemed transplantable, they may be matched to one of the available organs, which are added at each time step of the simulation. For candidates who receive a transplant, post-transplant graft and patient survival are predicted. Candidates who are not transplantable or not transplanted during the current time step remain in the model, and time is advanced. At each new time point, candidates may be removed from the waiting list due to death or becoming permanently unfit for transplantation. New candidates are also added to the waiting list at every step. The simulation is run until the specified number of time steps is reached. Images provided by Servier Medical Art (https://smart.servier.com/), licensed under CC BY 4.0 (https://creativecommons.org/licenses/by/4.0/).

The general model workflow is presented in Figure 1 (and in more detail in Supplementary Figure S1). Rare or highly complex processes were simplified to balance model stability and to focus on the core allocation dynamics.

#### Initialization

The simulation begins with a predefined waiting list, encompassing all kidney transplant candidates listed in the German national transplant registry as of January 1, 2006.

Each candidate on the initial waiting list has the following attributes: age, time since waiting list registration, time since start of dialysis, blood group, HLA type, DSO region, candidate status (transplantable or not transplantable), latest percentage of virtual Panel Reactive Antibodies (vPRA) (the term utilized by Eurotransplant for calculated PRA) and latest unacceptable antigens.

Missing HLA entries are imputed by resampling from HLA haplotype frequencies (see SDC Methods, HLA Types).

#### Synthetic candidate generation

Newly registered candidates are periodically added to the waiting list. Their attributes are generated in two stages.

##### Core demographic and immunologic attributes

Age at registration, blood group, and DSO region are independently resampled from the preprocessed kidney waiting list of the German national transplant registry (see SDC Methods for details), while HLA types are sampled independently from population HLA haplotype frequencies (see SDC Methods, HLA type).

Potential correlations between age at registration, DSO region, and blood group were evaluated using chi-squared, Kruskal-Wallis, Cramér’s V, and eta-squared tests; all detected associations were weak. Given the limited strength of these associations and the focus on preserving the key marginal distributions, independent resampling was considered an appropriate approach.

##### Conditionally generated clinical and immunological attributes

Conditional on the core attributes, the following attributes are generated:

Dialysis to registration time, using age-stratified Gaussian kernel density estimation (see SDC Methods, Dialysis to Waiting list registration time).

Unacceptable antigens and vPRAs are sampled from empirical vPRA strata and filtered to avoid inconsistency with candidate’s HLA type (see SDC Methods, vPRA).

#### Assigned allocation relevant and dynamic attributes

Each candidate–whether part of the initial cohort or synthetically added–is assigned the following attributes: ET allocation program (ETKAS or ESP), living donor donation probability, mismatch probability points, DSO subregion (see SDC Methods for details) and predicted time for removal from the waiting list (derived from a Cox proportional hazards model; see SDC Methods, Removal Model). Candidate status within the model can take four states: transplantable, not transplantable, living donor transplanted, or removed.

Living donor donations are predicted for candidates who have been on the waiting list for less than 1 year, using age-specific probabilities derived from observed living donor donation rates in 5-year age groups (Supplementary Figure S2A; see SDC Methods, Living Donor Probability).

Whether a candidate is currently considered transplantable or not is determined using a multistate recurrent event framework, with transition probabilities estimated via the Aalen-Johansen estimator (Supplementary Figure S3; see SDC Methods, Transplant Status).

#### Donor attributes

Organ donors are added based on the annual number of transplanted deceased donor kidneys (Supplementary Table S1). Donor attributes include age, blood group, HLA type, DSO region, and subregion. Except for HLA types, all donor attributes are independently resampled from historical deceased donor kidney donations in the registry (see SDC Methods for details). HLA types are resampled from published estimated four loci haplotype frequencies derived from the DKMS [25] (see SDC Methods, HLA Types).

#### Allocation

At each discrete simulation step, newly introduced donor organs are allocated to transplantable candidates on the waiting list. Allocation follows the ET allocation rules, which were modified in parts for simplification (Supplementary Figure S4; see SDC Methods, Allocation). Organ offer acceptance is simulated by a piecewise logistic regression model incorporating key predictors of acceptance, with vPRA, recipient age, donor age, years on dialysis and the number of HLA mismatches (see SDC Methods, Organ Acceptance; Supplementary Tables S2, S3). Organs that cannot be matched within a given step are removed from the model.

#### Time progress and removal from waiting list

After allocation, the simulation time advances by the predefined step size. The model then checks whether a candidate should be removed from the waiting list based on his/her simulated survival time, generated from a Cox proportional hazards model with a parametric baseline hazard, estimated using natural cubic splines with four knots, placed at equally spaced time points, fitted to the German national registry data (Supplementary Figure S5). Left censoring was applied to candidates registered before January 1, 2006, and transplantation events were treated as right-censoring. Proportional hazards assumptions were evaluated using Schoenfeld residual–based tests and graphical diagnostics implemented in the *lifelines* package. Observed violations in the dialysis to registration time were addressed through square-root transformation and age effects were handled using age-stratified baseline hazards.

Removal times are simulated using inverse hazard sampling as adapted from Bender et al. [28] (see SDC Methods, Removal Model).

Relisting is approximated by reintroducing candidates as newly generated individuals, which is considered by including relisted candidates in the sampling space and the total count of new registrations.

After each time step, new synthetic candidates are added to the waiting list according to the specified annual number of new registrations (Supplementary Table S4), which were evenly distributed across time steps. The simulation continues until a pre-defined number of steps has been reached.

### Verification and validation

Before building the model, we validated the registry data through consistency checks, outlier detection and comparison to annual published reports by ET and the DSO [23, 24]. For model verification, we employed trace analysis (following individual simulated cases to ensure logical progression), testing under extreme and degenerate conditions, examining input-output relationships, and reprogramming critical components to cross-check results [29]. Reprogramming was applied in particular for the removal prediction component.

For model validation, we employed face validity (assessment by clinical experts), internal validity (evaluating stability of results across repeated simulations), analysis of input-output relationships, and graphical comparisons of simulated outcomes with registry data [29].

### “ESP omitted” scenario

To demonstrate how different allocation strategies could be simulated by the model, we compared the current allocation rules with an alternative model that omitted ESP. Both simulations were run five times. The simulations covered the period from January 1, 2006, to January 1, 2017. In the “ESP omitted” scenario, organ allocation was performed exclusively through ETKAS. For simulating organ acceptance from donors aged ≥65 years, we applied the odds ratios from ETKAS. This assumption reflects current ETKAS practice, in which kidneys from older donors may be allocated through rescue mechanisms. All other parameters remained unchanged.

### Software

Data processing and preparation were performed in R (version 4.5.0) [30], using the packages *tidyverse* (version 2.0.0) [31] and *survival* (version 3.8-3) [32] among others (Supplementary Table S5).

The simulation model was implemented in Python (version 3.11) using *Mesa* (version 2.2.4) [33]*, NumPy* (version 1.26.3) [34]*, pandas* (version 2.1.4) [35]*, SciPy* (version 1.11.4) [36], and *lifelines* (version 0.28.0) [37] among others (Supplementary Table S5). Parallel processing (via Python’s multiprocessing module) was used to reduce the total running time.

All code for model simulation is openly available on Github (https://github.com/na55imK/kidney_dtsim).

### Statistics

The model was run five times. For comparison between scenarios, the identical sequence of random numbers was used across different scenarios (common random numbers). Outcomes were reported as the median of these five runs and the minimum and maximum.

## Results

### Model validation

By January 1, 2017, the simulation showed 19,517 transplants [min-max; 19,516–19,517], matching the registry [19,517] (median deviation 0.00%).

**FIGURE 2:**
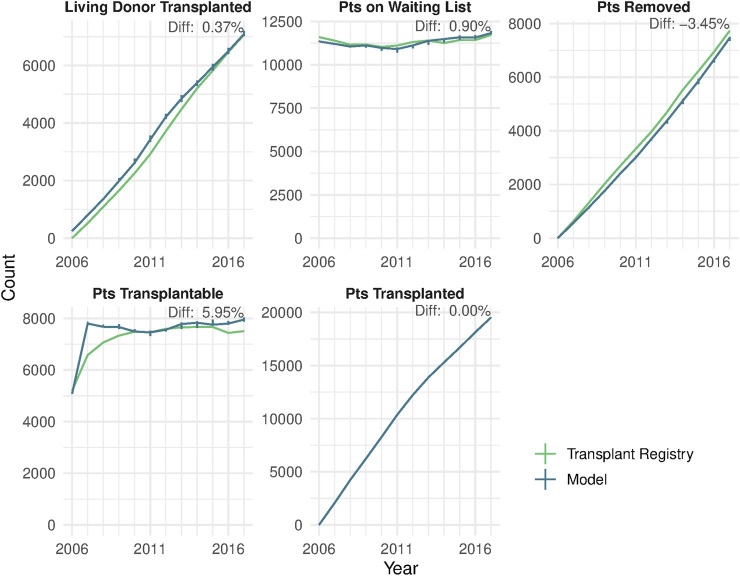
Comparison of waiting list dynamics and transplantations between the discrete time simulation and the data from the German national transplant registry. Median percentage deviation of the discrete time simulation from the German national transplant registry is shown as of January 1, 2017, with bars indicating the minimum and maximum. Living donor transplanted, removed patients, and transplanted patients show the cumulative count since January 1, 2006. Abbreviations: Pts, patients; Diff, difference.

The median number of candidates on the waiting list was 11,828 [11,758–11,963] at the end of the simulation period, while 11,722 were recorded in the registry (median deviation 0.90%). The number of transplantable candidates differed slightly more, with a model median of 7,948 [7,874–8,029] versus 7,502 in the registry (median deviation 5.95%). The cumulative number of candidates who left the waiting list due to death or deteriorated health status was slightly underestimated by the model, with a deviation of −3.45% (model median 7,461 [7,356–7,506]; registry 7,728). Kaplan-Meier curves for waiting list removals generated by the model closely resembled those from the registry (Supplementary Figure S6). Living donor donations showed a deviation of 0.37% (model median 7,090 [7,031–7,204]; registry 7,064) (Figure 2).

**FIGURE 3:**
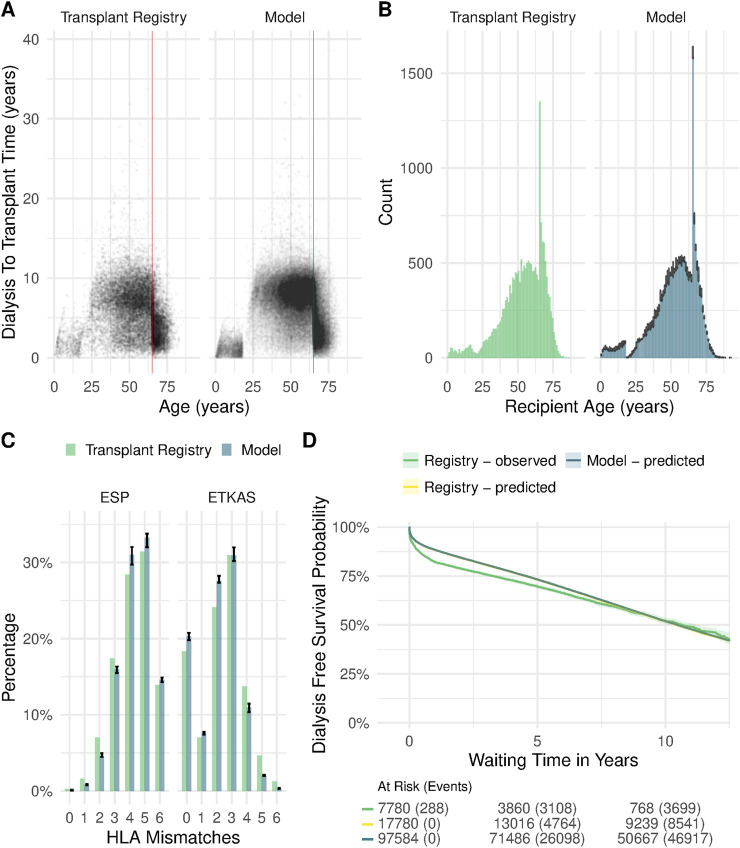
Proof of model reliability through comparison of multiple measures with historical data from the German national transplant registry **(A)** Dialysis to transplant time in years for all transplanted patients is plotted against age at transplantation in years. Every dot corresponds to one transplantation. For the discrete time simulation model, data were pooled from five independent model runs. **(B)** Recipient age at transplantation distribution grouped into one-year bins. For the simulation, the median across five model runs is shown, with black bars representing the minimum and maximum. **(C)** Distribution of HLA ABDR mismatches, shown as the percentage of transplants for each mismatch count. For the simulation, the median across five model runs is shown, with black bars indicating the minimum and maximum. Data are displayed separately for transplants in the ETKAS and the ESP. **(D)** Dialysis free survival probability is displayed. The observed dialysis free survival from the registry data is shown as a Kaplan-Meier survival curve. Predicted survival was simulated using a Royston-Parmar model published by Coemans et al., once using data from the transplant registry and once using data from the five runs of the model. Abbreviations: ESP, European Senior Program; ETKAS, Eurotransplant Kidney Allocation System; HLA, Human Leukocyte Antigen.

Visual inspection of dialysis-to-transplant time (Figure 3A) and waiting list-to-transplant time (Supplementary Figure S7A) showed good alignment between the combined model runs and historical data, clearly separating the following groups: pediatric, 18–64 years, and 65 years and older. The median dialysis-to-transplant time was 6.95 years [IQR 4.12–8.73] in the model and 5.81 years [IQR 3.27–7.91] in the registry (Supplementary Table S6). The model demonstrated good stability across individual runs (Supplementary Figures S7C,D).

The distribution of recipient age at transplant showed the characteristic peak [1] at around 65 years. Recipients aged 65 years and pediatric recipients were slightly overrepresented in the model, whereas recipients aged older than 65 years were slightly underrepresented (Figure 3B). The median recipient age for ETKAS was 51 years [IQR 42–59] in the model and 51 years [IQR 42–59] in the registry. For the ESP, the median recipient age was 68 years [IQR 66–71] in the model and 68 years [IQR 66–71] in the registry (Table 1). The age gap (recipient age - donor age) was aligned well between model and registry data (Table 1; Supplementary Figure S7B).

**TABLE 1:** Comparison of model outcomes with data from German national transplant registry.

Characteristic	Transplant Registry			Model	
	AM N = 526	ESP N = 4,765	ETKAS N = 14,209	ESP N = 23,494	ETKAS N = 74,090
Age gap	1 (−9, 12)	−3 (−7, 1)	0 (−10, 10)	−3 (−7, 0)	0 (−11, 12)
Unknown	169	0	1,564	​	​
Age donor	49 (40, 57)	72 (68, 75)	51 (42, 58)	71 (68, 75)	51 (42, 58)
Unknown	169	0	1,564	​	​
Age recipient	48 (40, 56)	68 (66, 71)	51 (42, 59)	68 (66, 71)	51 (42, 59)
Recipient age group					
<18	1.5%	0.0%	5.1%	​	6.4% [6.1%–6.5%]
18-64	92.0%	0.0%	88.6%	​	87.6% [87.5%–87.7%]
≥65	6.5%	100.0%	6.3%	100.0% [100.0%–100.0%]	6.1% [5.8%–6.3%]
Donor age group					
<18	2.8%	0.0%	4.5%	​	4.4% [4.0%–4.5%]
18-64	96.9%	0.0%	90.0%	​	89.8% [89.6%–90.7%]
≥65	0.3%	100.0%	5.4%	100.0% [100.0%–100.0%]	5.8% [5.1%–6.2%]
Unknown	169	0	1,564	​	​
Recipient blood group					
A	45.4%	44.6%	45.2%	45.4% [44.7%–46.4%]	43.5% [43.2%–43.8%]
AB	5.5%	3.8%	6.6%	2.6% [2.5%–2.9%]	5.7% [5.3%–5.9%]
B	13.9%	11.6%	12.1%	9.5% [9.1%–9.9%]	11.9% [11.7%–11.9%]
O	35.2%	40.0%	36.1%	42.2% [41.6%–43.0%]	39.1% [38.4%–39.3%]
Donor blood group					
A	30.3%	43.0%	44.8%	45.4% [44.7%–46.4%]	43.5% [43.2%–43.8%]
AB	0.0%	2.5%	6.0%	2.6% [2.5%–2.9%]	5.7% [5.3%–5.9%]
B	3.9%	10.7%	11.7%	9.5% [9.1%–9.9%]	11.9% [11.7%–11.9%]
O	65.8%	43.8%	37.6%	42.2% [41.6%–43.0%]	39.1% [38.4%–39.3%]
Unknown	169	0	1,564	​	​
vPRA					
0%	6.1%	90.5%	82.6%	89.4% [89.1%–89.9%]	83.7% [83.5%–83.9%]
>0%-<85%	17.7%	8.7%	15.0%	9.6% [9.2%–10.1%]	12.6% [12.5%–12.9%]
≥85%	76.2%	0.8%	2.3%	1.0% [0.9%–1.1%]	3.7% [3.5%–3.7%]
HLA ABDR mismatches					
0	10.5%	0.3%	18.3%	0.1% [0.0%–0.1%]	20.3% [19.8%–20.8%]
1	21.9%	1.6%	7.0%	0.8% [0.8%–0.9%]	7.6% [7.4%–7.7%]
2	38.6%	7.0%	24.1%	4.6% [4.5%–5.0%]	27.6% [27.3%–28.2%]
3	24.8%	17.4%	30.9%	16.0% [15.5%–16.3%]	30.9% [30.2%–32.0%]
4	4.3%	28.4%	13.7%	31.0% [29.7%–32.0%]	10.9% [10.4%–11.5%]
5	0.0%	31.4%	4.6%	33.2% [32.0%–33.8%]	2.0% [2.0%–2.1%]
6	0.0%	13.9%	1.3%	14.6% [14.3%–14.9%]	0.3% [0.3%–0.4%]
Unknown	316	1,914	6,614	​	​
Recipient region					
Baden-Württemberg	11.8%	10.7%	10.7%	10.6% [10.2%–11.1%]	11.2% [11.1%–11.5%]
Bayern	10.2%	14.8%	15.1%	15.0% [14.5%–15.7%]	15.3% [14.9%–15.7%]
Mitte	13.4%	12.4%	13.7%	12.4% [11.5%–12.5%]	12.2% [11.8%–12.3%]
Nord	17.7%	16.5%	15.9%	16.9% [16.5%–17.4%]	16.7% [16.5%–16.8%]
Nord-Ost	11.4%	10.2%	9.1%	11.3% [10.9%–11.4%]	11.3% [10.9%–11.4%]
Nordrhein-Westfalen	23.2%	22.3%	24.2%	23.2% [23.0%–23.2%]	21.7% [21.2%–21.7%]
Ost	12.4%	13.1%	11.4%	10.6% [10.5%–11.5%]	11.9% [11.6%–12.1%]
Unknown	17	23	161	​	​
Donor region					
Baden-Württemberg	13.4%	11.0%	11.3%	10.6% [10.2%–11.1%]	11.6% [11.4%–11.7%]
Bayern	16.5%	14.7%	15.7%	15.0% [14.5%–15.7%]	15.7% [15.4%–16.1%]
Mitte	11.6%	10.9%	12.2%	12.4% [11.5%–12.5%]	11.7% [11.3%–12.2%]
Nord	19.7%	17.1%	16.2%	16.9% [16.5%–17.4%]	16.6% [16.5%–16.8%]
Nord-Ost	10.9%	11.7%	11.2%	11.3% [10.9%–11.4%]	11.3% [11.0%–11.4%]
Nordrhein-Westfalen	18.3%	22.6%	20.8%	23.2% [23.0%–23.2%]	20.2% [19.8%–20.5%]
Ost	9.5%	12.1%	12.6%	10.6% [10.5%–11.5%]	12.8% [12.5%–13.3%]
Unknown	242	3	2,294	​	​
Donor location relative to recipient					
Abroad	46.0%	0.1%	16.1%	​	​
Home country	46.4%	9.8%	23.5%	​	19.1% [18.9%–19.7%]
Regional	7.6%	90.2%	60.5%	100.0% [100.0%–100.0%]	80.9% [80.3%–81.1%]

For the model, N equals the number of cases across all pooled simulations.

For continuous variables, the median (IQR) is calculated across all pooled simulations. For categorical variables, the percentage is first calculated within each model run, and then the median [min./max.] across model runs is reported.

Abbreviations: AM, acceptable mismatch; ESP, european senior program; ETKAS, eurotransplant kidney allocation system; HLA, human leukocyte antigen; vPRA, virtual Panel Reactive Antibodies.

Distribution of HLA mismatches in the model showed a similar pattern to that observed in the registry for ESP and ETKAS recipients (Table 1; Figure 3C).

Validity of the sub model for post-transplant risk prediction (published by Coemans et al. [38]) was evaluated by comparing model-predicted survival with outcomes observed in the transplant registry. The registry data showed a steeper decline in the Kaplan-Meier curve for dialysis-free survival probability, particularly within the first 2.5 years after transplantation, with both curves converging at approximately 10 years post-transplant (Figure 3D), indicating long-term post-transplant risk profiles generated by the model were similar to those observed in the registry data. Overall survival showed a similar pattern (Supplementary Figure S8A). Graft survival probability, estimated using an Aalen-Johansen estimator, also showed an initially steeper decline followed by a slower decline (Supplementary Figure S8B).

### Simulated impact of omitting ESP

**FIGURE 4:**
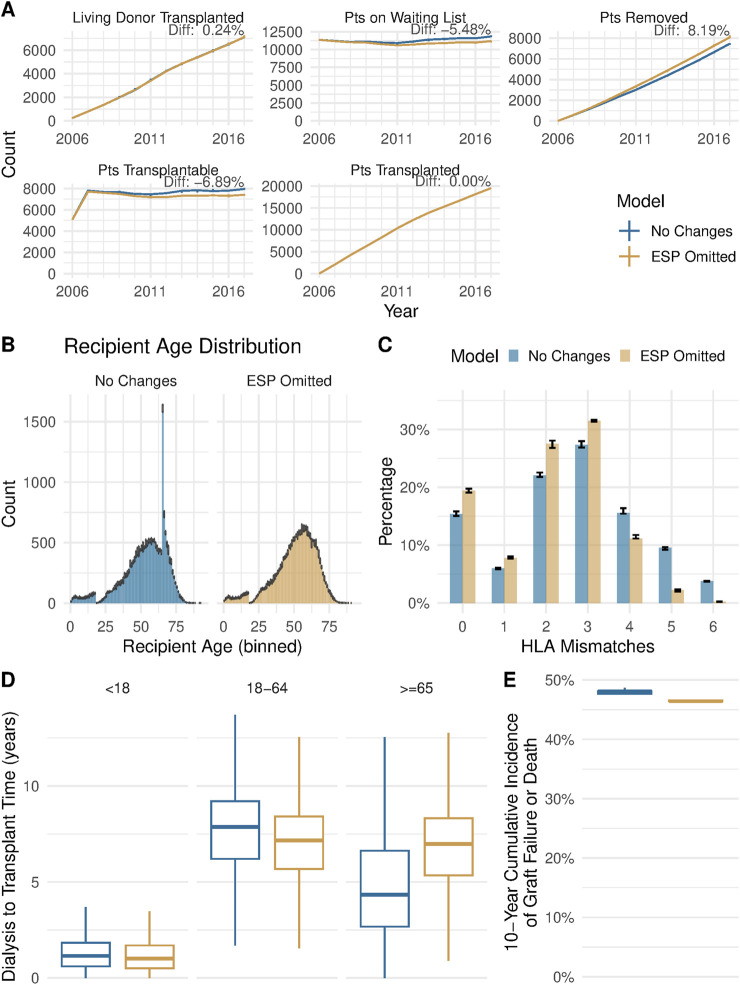
Scenario analysis of omitting the ESP. Two different scenarios were compared; simulation of the Eurotransplant allocation rules, as currently in place (“no changes”), and simulation of a scenario where only the ETKAS rules are used for allocation (“ESP omitted”). **(A)** Evolution of the waiting list and the number of transplantations over the simulated time frame. Median percentage deviation of the “ESP omitted” scenario from the “no changes” scenario is shown as of January 1, 2017. Living donor transplanted, removed patients, and transplanted patients show the cumulative count since January 1, 2006. **(B)** Age distribution of transplant recipients. Age was grouped into one-year bins. The median across five model runs per scenario is displayed, with black bars indicating the minimum and maximum. **(C)** Distribution of HLA ABDR mismatches, displayed as the percentage of transplants by mismatch count. The median across five model runs per scenario is displayed, with black bars indicating the minimum and maximum. **(D)** Boxplot of dialysis to transplant time, stratified by recipient age group across both scenarios. **(E)** Boxplot of predicted 10-year cumulative incidence of graft failure or death across both scenarios. Cumulative incidences were simulated based on a Royston-Parmar model published by Coemans et al. [38]. Abbreviations: Pts, patients; Diff, difference; ESP, European Senior Program; ETKAS, Eurotransplant Kidney Allocation System; HLA, Human Leukocyte Antigen.

At the end of the simulation period, the median number of patients on the waiting list was slightly lower in the “ESP omitted” model with 11,180 [11,040–11,214] patients compared to the “no changes” model with 11,828 [11,758–11,963] patients. Similarly, the median number of transplantable patients was lower in the “ESP omitted” model (7,400 [7,337–7,475]) than in the “no changes” model (7,948 [7,874–8,029]). These differences were driven by a higher number of candidates leaving the waiting list due to death or permanent unfitness for transplantation in the “ESP omitted” model (8,072 [8,017–8,260]) compared to the “no changes” model (7,461 [7,356–7,506]) (Figure 4A). There was little variation in the number of transplantations, as the model was constructed exclusively using the characteristics and quantities of transplanted organs.

Omitting ESP resulted in the disappearance of the spike at age 65 among organ recipients (Figure 4B). In addition, better overall minimization of HLA mismatches was observed (Figure 4C; Supplementary Table S7), e.g., zero mismatch allocation increased from 15.4% [15.0%–15.8%] to 19.4% [19.1%–19.8%] and 6/6 mismatch allocation decreased from 3.8% [3.7%–3.8%] to 0.2% [0.2%–0.3%] (Supplementary Table S7). In the “ESP omitted” model, 16.1% [16.0%–16.5%] of organs were transplanted into recipients aged 65 years and older, compared to 28.7% [28.4%–29.0%] in the “no changes” model (Supplementary Table S7). Dialysis-to-transplant time increased for recipients aged 65 years and older when ESP was omitted, with a median of 6.97 years [IQR 5.35–8.32], compared to 4.33 years [IQR 2.67–6.63] in the “no changes” model. Among recipients aged 18–64 years, the median waiting time decreased modestly (“ESP omitted”: 7.17 years [IQR 5.67–8.42]; “no changes”: 7.86 years [IQR 6.20–9.20]) (Figure 4D; Supplementary Figure S9A).

When ESP was omitted, recipients were on average younger relative to their donors, with a median age difference of −3 years [IQR -16–10], compared to −1 year [IQR -10–8] in the “no changes” model (Supplementary Table S7; Supplementary Figure S9B). Regional organ allocation decreased slightly in the “ESP omitted” model, with a median of 81.8% [81.5%–82.1%], compared to 85.6% [85.0%–85.6%] in the “no changes” model. Despite this shift, the distribution of organ recipients across regions remained nearly unchanged (Supplementary Table S7).

The median predicted 10-year cumulative incidence of the composite outcome of graft failure or death was 46.4% [IQR 46.4%–46.4%] in the “ESP omitted” model compared with 48.1% [IQR 47.7%–48.1%] in the “no changes” model (Figure 4E). The 10-year cumulative incidence of death with a functioning graft was likewise lower in the “ESP omitted” model, with 24.3% [IQR 24.2%–24.4%], compared to 28.3% [IQR 28.1%–28.3%] in the “no changes” model (Supplementary Figure S10C). In contrast, the predicted 10-year cumulative incidence of graft failure alone was slightly higher in the “ESP omitted” model with 28.2% [IQR 28.0%–28.2%], compared to 27.7% [IQR 27.5%–27.7%] (Supplementary Figure S9E).

## Discussion

We developed a novel patient-level simulation model based on the ET kidney allocation algorithm. More than 35,000 patient-level entries from the German transplant registry were utilized, supplemented by data from DKMS and ET. The model incorporates multiple stochastic sub models to simulate key aspects of the transplant process, including candidate listing status, waiting list removal, organ acceptance, and post-transplant risk. Over an 11-year simulation period, the model closely reproduced observed dynamics in waiting list size, transplantation rates, and patient removals. While a brief burn-in period was observed—particularly in the number of transplantable candidates during the first two years—the model demonstrated high concordance with the observed patient, donor, and match characteristics.

We demonstrated the model’s adaptability and capacity by simulating a policy scenario in which the ESP was omitted. This scenario highlighted the ESP’s role for candidates aged over 65, as its removal resulted in increased waiting times in this age group and higher waiting list removals.

Allocation simulation models are inherently system-specific, as they are designed to replicate the rules, and constraints of a given allocation system. As applied in this study, calibration using historical data and validation by comparison with observed system behavior are standard approaches in this field [10, 17, 29, 39–42].

Despite the recognized value of simulated allocation models, few exist for the ET area. Existing models show notable limitations. One model simulates the matching process at a more or less static time point [16], restricting its ability to assess changes in waiting list dynamics or transplant activity over time. Another model focuses on a small population within a kidney exchange program [17]. The model by Niemann et al. [10] explores the feasibility of epitope matching within ETKAS, offering a broader scope but excluding the ESP, which accounts for approximately one-quarter of kidney transplants in the region. Unlike the Niemann model, our simulations also incorporate living donor transplants, which were not part of their framework. In Niemann et al [10] candidate characteristics were drawn from the 2015 ET annual report, with random waiting list removals and organ acceptance fixed at a 10% probability. In contrast, our model is fitted to patient-level data and incorporates stochastic submodels for waiting list removals and organ acceptance, allowing a more dynamic and realistic simulation. Compared to the ETKidney simulator [21] - a discrete event simulation model currently under development and based directly on data from ET–our model does not require complete longitudinal datasets. It requires patient level data only for the initial waiting list, while all other inputs can be provided as summary statistics or empirical lists (e.g., list of donor ages or HLA types), facilitating flexible adjustment of model parameters and enabling exploration of future allocation dynamics through extrapolated input parameters; additionally, these inputs can be shared with minimal privacy concerns. The ETKidney Simulator [21] also does not consider living donor transplants.

Validation over more than a decade enables assessment of long-term impacts, whereas most published models cover 4 years or less [10, 40] or rely on static designs [16]. Despite operating at the patient-level, our model remains computationally efficient, completing in under an hour on a personal computer.

All simulation models require explicit assumptions about system behavior, reflecting an inherent trade-off between capturing sufficient complexity and maintaining robustness. When a model becomes too complex, the reliability of scenario analyses can be reduced, as it limits how confidently results can be generalized to different settings. Accordingly, several simplifications were made during development. The Acceptable Mismatch (AM) program was not implemented, as it accounted for only 2.7% (n = 526) of transplantations between 2006 and 2017; these candidates were instead allocated through ETKAS and ESP. Similarly, “high urgent” status was not implemented (n = 332, 1.7% of transplants). Multiorgan transplants were also not implemented. Candidates listed for multiple organs would have different model behavior (e.g., in acceptance, waiting list survival etc.), making the model far more complex with limited benefit for the study objectives.

Model accuracy is impaired for subgroups with limited available data, such as very old or very young candidates and donors. It could be expanded, with available data, to other countries in the ET region and the time after 2016. Direct application of the model to allocation systems outside the ET region is limited, but the methodological framework is transferable and can be adapted to other regions. Improvements of the model could be made with better data regarding HLA types, vPRAs and follow-up, which are currently limited in quality in the German national transplant registry.

In conclusion, our work enables policymakers to assess both system-level and individual consequences, such as shifts in transplant rates, waiting list dynamics, or post-transplant risks, before implementing changes in practice. It also offers the opportunity to retrospectively reassess past policy decisions in the ET-area. Moreover, the simulator could estimate the impact of changing external conditions, such as an aging society, declining or rising donor rates.

## Data Availability

The datasets presented in this article are not readily available because the registry data analyzed in this study contains anonymized, patient-level information and therefore cannot be made publicly available. Access may be granted by the German national transplant registry upon reasonable request. Aggregated model input data and the simulation model are publicly available at https://github.com/na55imK/kidney_dtsim. Requests to access the datasets should be directed to https://transplantations-register.de.
