# Correction: A discrete time simulation model for kidney allocation in Germany

**DOI:** 10.3389/ti.2026.17122

**Published:** 2026-06-23

**Authors:** Transplant International Production Office

**Affiliations:** Frontiers Media SA, Lausanne, Switzerland

**Keywords:** discrete time simulation, Eurotransplant, Germany, kidney allocation, simulated allocation models

Author Roland Schmitt was erroneously assigned as a senior author.

The original article has been updated.

