# Supplementary material for: A discrete time simulation model for kidney allocation in Germany
Source: Transpl Int. 2026 May 28;39:16189. doi: 10.3389/ti.2026.16189 (PMC13253491; doi:10.3389/ti.2026.16189)
Supplement: Supplementary file 1 [file DataSheet1.pdf]

Supplemental Digital Content (SDC)

Table of contents

Supplemental Methods *2*
HLA Types 2
Virtual Panel Reactive Antibodies (vPRA) 2
HLA Frequencies 3
Age at Waiting List Registration and Donor Age 3
Dialysis to Waiting List Registration Time 3
Blood Group and Region 3
Living Donor Probability 4
Eurotransplant Programs 4
Removal Model 4
Transplant Status Prediction 5
Organ Acceptance 5
Allocation 5
Post Transplant Survival 6
Supplemental References *7*
Supplemental Tables *9*
Table S1: Number of deceased donor transplantations per year used as model Input 9
Table S2: Odd ratios for organ acceptance in the ETKAS program 9
Table S3: Odds ratios for organ acceptance in the ESP 10
Table S4: Number of waiting list registrations per year used as model Input 10
Table S5: Software environments and packages used in data processing and simulation 11
Table S6: Comparison of waiting times between model and national registry data 11
Table S7: Scenario analysis of omitting the ESP 12
Table S8: Frequency of regions used as model input for candidates 14
Table S9: Frequency of blood groups used as model input for candidates 14
Table S10: Frequency of regions used as model input for organs 14
Table S11: Frequency of blood groups used as model input for organs 14
Supplemental Figures *15*
Figure S1: Flowchart of the discrete-time simulation model for organ allocation and transplantation 15
Figure S2: Input parameters for the simulated allocation model 16
Figure S3: Estimation of transplantability status used inside the simulation model 18

Figure S4: Flowchart of organ allocation in the model **19**

Figure S5: Estimation of removal from the waiting list within the simulation model. **20**

Figure S6: Comparison of waiting list removals between model data and data from the German national transplant registry **21**

Figure S7: Validation of model reliability trough comparison with historical data from the German national transplant registry **23**

Figure S8: Post transplant model behavior **25**

Figure S9: Comparison of waiting times and age gaps between both scenarios......................... **26**

Figure S10: Analysis of post-transplant outcome under both allocation scenarios **27**

Supplemental Methods

HLA Types

Human Leukocyte Antigen (HLA) types are derived from published four-locus haplotype frequency estimates based on data from the German bone marrow donor registry (DKMS) (1). The loci considered were HLA-A, HLA-B, HLA-C, and HLA-DRB1. For loci HLA-A, HLA-B, HLA-C, and HLA-DRB1, the corresponding Eurotransplant (ET) match determinant was obtained from the ET Reference Laboratory website (2).

In cases where HLA types were partially reported or missing in the waiting list data, imputation was performed. If partial HLA type information and unacceptable antigens were present, the haplotype frequencies were filtered to match the reported alleles and exclude haplotypes containing the unacceptable antigens. Some partially reported antigen combinations were not covered by the haplotype frequency dataset. In such cases, imputation was performed without filtering for the already reported loci.

For transplant candidates added after the initial waiting list, HLA haplotypes at the split antigen level for loci A, B, C, and DRB1 are resampled from the above-mentioned haplotype frequencies.

Virtual Panel Reactive Antibodies (vPRA)

In the German national registry, virtual Panel Reactive Antibodies (vPRA) values are not available for all cases. For such cases, the ET Reference Laboratory's Virtual PRA calculator (3) was used. vPRAs are calculated by ET based on unacceptable antigens. The ET Reference Laboratory uses a database with 10,000 individuals from the ET area, provided by the DKMS.

For candidates on the initial waiting list, the most recent vPRA measurement prior to transplantation was used. The empirical frequency of unacceptable antigen sets and their corresponding vPRA values were determined by using all entries in the registry for kidney waiting list candidates.
For newly generated candidates, unacceptable antigens and their corresponding vPRA values are assigned by first stratifying the sampling space into vPRA groups (0%, 0%–49%, 50%–84%, and ≥84%), preserving the empirical proportions observed in the registry. Within each stratum, unacceptable antigens are sampled according to their empirical frequencies, with adjustments to exclude any profiles that conflict with the candidate's own HLA alleles.

HLA Frequencies

For calculation of the mismatch probability points, the allele frequencies are needed. For this purpose, we used the "Germany pop 8" dataset from the Allele Frequency Net Database (4). This dataset included 39,698 individuals, with samples sourced from the DKMS and uploaded by J. Mytilineos (5). The Eurotransplant Reference Laboratory HLA tables were used to map HLA-A and HLA-B alleles to their corresponding broad antigens and HLA-DR alleles their corresponding split antigens (2).

Age at Waiting List Registration and Donor Age

For all newly generated candidates on the waiting list, age at waiting list generation is resampled using a Gaussian kernel density estimation (KDE). This non-parametric approach was chosen because it flexibly models the empirical age distribution without assuming a specific parametric form. Bandwidth was determined using Scott's rule. A list of reference ages must be provided as model input. We used all candidates from the preprocessed kidney waiting list of the German national transplant registry registered after January 1, 2006, for this purpose (Figure S2B).

Similarly, donor ages are resampled using a Gaussian KDE. A list of donor ages must also be provided as input; we used all deceased donor kidney records from the registry (Figure S2C). Some donor records had missing age information. Upon inspection, these cases were found to predominantly involve donors from abroad. As most of these organs were transplanted in the Eurotransplant Kidney Allocation System (ETKAS) program, missing donor ages were imputed by randomly sampling from the observed age distribution of ETKAS donors.

Negative values for donor or candidate age, which can arise due to the KDE resampling process, were discarded and resampled until a valid positive age was obtained.

Dialysis to Waiting List Registration Time

The time from dialysis initiation to waiting list registration was simulated for newly generated candidates. This required a data frame containing both the time from dialysis registration and the corresponding binned age at registration. These data were derived from the national registry, with filtering for candidates registered on the waiting list after January 1, 2006. Cases with missing time values were excluded, primarily involving candidates who were preemptively listed and either removed from the waiting list or transplanted before dialysis initiation, resulting in undefined dialysis-to registration intervals.

Age at registration was stratified into 10-year age groups. Within the model, a time value for each candidate was resampled from a Gaussian KDE corresponding to their age group (Figure S2D). Negative resampled values were set to zero.

Blood Group and Region

Blood group and German Organ Procurement Organization (DSO) region for new waiting list candidates are sampled using distributions provided as model input (Tables S8-S9). These distributions were derived from all candidates on the cleaned waiting list registered after January 1, 2006.

Similarly, blood group and DSO region for organ donors are sampled using distributions calculated from the deceased kidney donor data (Tables S10-S11).

Because the transplant registry does not provide subregion information for either donors or candidates, individuals are randomly assigned to one of the subregions within their respective DSO region.

Living Donor Probability

To account for living donor transplants among newly registered candidates, the model incorporates observed living donor donation rates. All candidates newly registered on the waiting list between January 1, 2005, and January 1, 2016, were considered from the registry data. Candidates were classified into registration periods: before 2006, 2006 – 2008, 2009 – 2010, 2011 – 2012, and 2013 onward. Within each period, candidates were further stratified into five-year age groups.

The resulting living donor rates were used as probabilities for assigning living donor transplants to newly added candidates within the model. For candidates already present on the waiting list at the start of the simulation, only those with a waiting time less than one year were considered for living donor donations.

Eurotransplant Programs

Within the simulation, waiting list candidates have a 36% probability of remaining in the ETKAS program after turning 65 years of age. This probability is calculated as the proportion of ETKAS cases among all candidates under 65 years old at the time of registration who have a reported value for the allocation program variable in the registry's waiting list data.

Unlike in other ET countries, where candidates are required to transfer into the European Senior Program (ESP) upon turning 65, candidates in Germany may continue to participate in ETKAS if eligible.

Removal Model

Removal from the waiting list was simulated using a Cox proportional hazards model with a parametric baseline hazard, estimated using natural cubic splines with four knots placed at equally spaced time points. Proportional hazards assumptions were assessed based on scaled Schoenfeld residuals, via the `check_assumptions()` function from the *lifelines* Python package. We examined both the resulting test statistics and the corresponding residual plots. The untransformed dialysis-to-registration time variable showed evidence of deviation from proportional hazards, which improved after applying square-root transformation. To allow for the square root transformation, values of zero or less were set to one. Age was handled by stratification into the following groups: 0–29, 30–39, 40–44, 45–49, 50–54, 55–59, 60–64, 65–69, 70–74, 75–79, and 80 years and older.

The model was fitted using waiting list data from the national transplantation registry. Left censoring was applied for candidates who entered the waiting list prior to January 1, 2006; consequently, their survival analysis was conditional on having already survived until the start of the study period. Patients were right censored at the time of transplantation, as transplantation represents a competing event that precludes removal from the waiting list via death. For patients who were neither transplanted nor removed from the waiting list, follow-up time was calculated as the duration from their registration date to January 1, 2017. Model calibration was evaluated by comparing observed and model-predicted cumulative removal curves (Figure S5).

For simulation of removal from the waiting list, for each candidate, a random number is drawn from a uniform distribution, applying the natural logarithm and identifying the time point at which the candidate's cumulative hazard function most closely matches that value. Adapted from the approach of Bender et al. (6) For candidates on the waiting list at model initialization the survival time is simulated on the condition of having already survived up to their waiting time at model entry.

Transplant Status Prediction

The status of a candidate on the waiting list was dynamically simulated at each step using an Aalen-Johansen estimator within a multistate recurrent event framework. The model was stratified by age at waiting list registration into the following groups: 0–32.4, 32.5–37.4, 37.5–42.4, 42.5–47.4, 47.5–52.4, 52.5–57.4, 57.5–62.4, 62.5–67.4, and 67.5 years and older. At each simulation step, the estimated probability of being non-transplantable at the specified time point for each age group was used to update candidate status. The model was fitted using data from the national registry (Figure S3).

Transplantations, removal from the waiting list, and death were treated as censoring events. Some candidates were missing an initial status. In such cases, the following assumptions were applied: if a subsequent status was available, it was assumed that the initial status matched the next recorded status for the candidate. If the next available status was censored and the initial status was missing, then: if a candidate was transplanted, the initial status was assumed to be transplantable; otherwise, it was assumed to be non-transplantable. Status updates occurring after censoring, due to transplantation or removal from the waiting list, were excluded, as only first-time listed cases were included in the analysis.
For candidates added at model initialization, the most recent recorded status on the waiting list prior to simulation start was used.

Organ Acceptance

When an organ was offered to a candidate, the model simulated acceptance using a piecewise logistic regression model. Odds ratios for organ acceptance in the ESP and ETKAS programs were required as model inputs. Because the German national registry does not provide data about organ acceptance and decline, the models were instead fitted using ET data. The following independent variables were used: vPRA, recipient age, donor age, presence of zero mismatches, and the number of mismatches at HLA-A, HLA-B, and HLA-DR loci (Tables S2-S3). While cold ischemia time and donor-transmitted infectious diseases play a significant role in clinical practice, they were not included as independent variables in the model, which represents a limitation of the current model.

Only kidneys placed through the regular ETKAS program or ESP were included in this analysis. Candidates whose profiles indicated that they did not wish to be offered the graft were excluded. Model fitting was based on ETKAS data from 2016 to 2020 and ESP data from 2014 to 2019.

Allocation

The allocation process in the simulation was based on the actual ET allocation policies (7), but implemented in a simplified form (see Figure S4). A simplified virtual crossmatch was performed by excluding donor-recipient pairs where the donor's HLA-A, HLA-B, HLA-C, or HLA-DRB1 antigens matched any of the recipient's unacceptable antigens, at either the split or broad level.

Organs could be offered up to 1000 times in one time step before being removed from the model.
High Urgency (HU) status was not simulated and thus not considered in allocation decisions. Additionally, the national kidney exchange balance between ET member countries was not incorporated, since allocation is only simulated in Germany. As a simplification of the extended and rescue allocation processes, kidneys that could not be allocated within the ESP were subsequently reallocated via the ETKAS program.

Post Transplant Survival

For transplanted patients, graft survival and patient death were simulated using the competing risks-corrected Royston-Parmar model published by Coemans et al. (8) The model was validated using data from the German national registry.

Follow-up data from the registry were processed as follows. Dates of death, follow-up, and graft failure recorded before the transplant date were set as not applicable. Organ failures recorded on the date of death were treated as censoring events. If both the follow-up date and the graft failure date were missing, the case was considered lost to follow-up on the transplantation date. Similarly, if the graft failure date occurred more than one year after transplantation and the follow-up date was missing, the case was also considered lost to follow-up on the transplantation date. The same approach was applied to the event of death.

Event times were simulated using the previously described approach by Bender et al. (6). Simulated event times exceeding 9997 days were treated as censored, as the Royston-Parmar model was only fitted up to 10,000 days and censoring avoided artificial inflation of event rates at the end of the observation period.

The observed cumulative incidence of dialysis-free survival was estimated using the Kaplan-Meier estimator. For graft survival and recipient survival, the competing risks Aalen-Johansen estimator was used.

Supplemental References

1. Seitz S, Lange V, Norman PJ, Sauter J, Schmidt AH. Estimating HLA haplotype frequencies from homozygous individuals – A Technical Report. International Journal of Immunogenetics. 2021;48(6):490–5.

2. Eurotransplant Reference Laboratory [Internet]. [cited 2025 Mar 30]. ETRL HLA tables. Available from: https://etrl.eurotransplant.org/resources/hla-tables/

3. Virtual PRA calculator [Internet]. [cited 2025 Mar 30]. Available from: https://www.etrl.org/vPRA.aspx

4. Gonzalez-Galarza FF, McCabe A, Santos EJMD, Jones J, Takeshita L, Ortega-Rivera ND, et al. Allele frequency net database (AFND) 2020 update: gold-standard data classification, open access genotype data and new query tools. Nucleic Acids Res. 2020 Jan 8;48(D1):D783–8.

5. The Allele Frequency Net Database [Germany pop 8] [Internet]. [cited 2025 Mar 30]. Available from: http://www.allelefrequencies.net/pop6001c.asp?pop_id=3089

6. Bender R, Augustin T, Blettner M. Generating survival times to simulate Cox proportional hazards models. Statistics in Medicine. 2005;24(11):1713–23.

7. Eurotransplant Manual – version 2025.2: Chapter 4 [Internet]. 2025. Available from: https://www.eurotransplant.org/wp-content/uploads/2025/03/H4-Kidney-2025.2-March-2025.pdf

8. Coemans M, Tran TH, Döhler B, Massie AB, Verbeke G, Segev DL, et al. A competing risks model to estimate the risk of graft failure and patient death after kidney transplantation using continuous donor-recipient age combinations. American Journal of Transplantation [Internet]. 2024 Aug 5 [cited 2024 Dec 7]; Available from: https://www.sciencedirect.com/science/article/pii/S1600613524004568

9. R Core Team. R: A language and environment for statistical computing [Internet]. Vienna, Austria; 2025. Available from: https://www.R-project.org/

10. Wickham H, Averick M, Bryan J, Chang W, McGowan L, François R, et al. Welcome to the Tidyverse. JOSS. 2019 Nov 21;4(43):1686.

11. Ushey K, Allaire J, Tang Y. reticulate: Interface to “Python” [Internet]. 2017 [cited 2025 Oct 20]. p. 1.43.0. Available from: https://CRAN.R-project.org/package=reticulate

12. Therneau TM. A Package for Survival Analysis in R [Internet]. 2024. Available from: https://CRAN.R-project.org/package=survival

13. Therneau TM, Grambsch PM. Modeling survival data: extending the Cox model. New York: Springer; 2001. 1 p. (Statistics for biology and health).

14. Sjoberg DD, Baillie M, Fruechtenicht C, Haesendonckx S, Treis T. ggsurvfit: Flexible Time-to-Event Figures [Internet]. 2022 [cited 2025 Oct 21]. p. 1.2.0. Available from: https://CRAN.R-project.org/package=ggsurvfit

15. Iannone R, Cheng J, Schloerke B, Hughes E, Lauer A, Seo J, et al. gt: Easily Create Presentation-Ready Display Tables [Internet]. 2020 [cited 2025 Oct 20]. p. 1.1.0. Available from: https://CRAN.R-project.org/package=gt

16. Sjoberg D D, Whiting K, Curry M, Lavery J A, Larmarange J. Reproducible Summary Tables with the gtsummary Package. The R Journal. 2021;13(1):570.

17. Wilke CO. cowplot: Streamlined Plot Theme and Plot Annotations for “ggplot2” [Internet]. 2015 [cited 2025 Oct 21]. p. 1.2.0. Available from: https://CRAN.R-project.org/package=cowplot

18. Müller K. here: A Simpler Way to Find Your Files [Internet]. 2017 [cited 2025 Oct 20]. p. 1.0.2. Available from: https://CRAN.R-project.org/package=here

19. Firke S. janitor: Simple Tools for Examining and Cleaning Dirty Data [Internet]. 2016 [cited 2025 Oct 21]. p. 2.2.1. Available from: https://CRAN.R-project.org/package=janitor

20. Ter Hoeven E, Kwakkel J, Hess V, Pike T, Wang B, Rht, et al. Mesa 3: Agent-based modeling with Python in 2025. JOSS. 2025 Mar 28;10(107):7668.

21. Harris CR, Millman KJ, Van Der Walt SJ, Gommers R, Virtanen P, Cournapeau D, et al. Array programming with NumPy. Nature. 2020 Sept 17;585(7825):357–62.

22. The pandas development team. pandas-dev/pandas: Pandas [Internet]. Zenodo; 2023 [cited 2025 Oct 21]. Available from: https://zenodo.org/doi/10.5281/zenodo.10304236

23. Virtanen P, Gommers R, Oliphant TE, Haberland M, Reddy T, Cournapeau D, et al. SciPy 1.0: fundamental algorithms for scientific computing in Python. Nat Methods. 2020 Mar 2;17(3):261–72.

24. Davidson-Pilon C. lifelines: survival analysis in Python. JOSS. 2019 Aug 4;4(40):1317.

25. The Matplotlib Development Team. Matplotlib: Visualization with Python [Internet]. Zenodo; 2025 [cited 2025 Oct 21]. Available from: https://zenodo.org/doi/10.5281/zenodo.592536

26. Waskom M. seaborn: statistical data visualization. JOSS. 2021 Apr 6;6(60):3021.

Supplemental Tables

Table S1: Number of deceased donor transplantations per year used as model Input

Year	Transplantations
2006	2067
2007	2167
2008	2004
2009	2028
2010	2092
2011	1866
2012	1658
2013	1417
2014	1385
2015	1450
2016	1383

Table S2: Odd ratios for organ acceptance in the ETKAS program

Abbreviations: HLA = Human Leukocyte Antigen; MM = mismatches; vPRA = virtual Panel Reactive Antibodies

Variable		Level	Beta Coef.	Odds Ratios
Intercept			0.236447228912349	1.26674070657144
vPRA			-0.0114982569818416	0.988567595337681
vPRA	Over 25		0.0155279688792177	1.01564915422959
vPRA	Over 50		-0.00226408988597392	0.9977384712323
vPRA	Over 85		-0.0451648856180989	0.955839864592172
Recipient Match Age			0.0133016014130455	1.0133904612688
Recipient Match Age	Under 25		-0.0046367141231214	0.995374018840839
Recipient Match Age	Over 50		0.00475128140812383	1.00476258664333
Recipient Match Age	Over 65		-0.038094166969919	0.962622289398909
Donor Age	Under 20		-0.0378847990485341	0.962823852726431
Donor Age			-0.0163379894899586	0.983794751569818
Donor Age	Over 65		-0.137984646834491	0.871112065970855
Zero MM		1	0.719045218819873	2.05247261259854
MM Broad HLA-A		1	-0.243818289851773	0.783630014852626
MM Broad HLA-A		2	-0.599215087433627	0.549242574346068
MM Broad HLA-B		1	-0.148486503142304	0.862011641540443
MM Broad HLA-B		2	-0.527400548919982	0.590137009871572
MM Split HLA-DR		1	-0.195810278313767	0.822168203020274
MM Split HLA-DR		2	-0.676810401712007	0.508235476842623
Years on Dialysis			0.0493913011880329	1.05063138362455

Table S3: Odds ratios for organ acceptance in the ESP

Abbreviations: HLA = Human Leukocyte Antigen; MM = mismatches; vPRA = virtual Panel Reactive Antibodies

Variable		Level	Beta Coef.	Odds Ratios
Intercept			1.35052966140309	3.85946920129482
vPRA			-0.0130995439215386	0.986985881684941
vPRA	Over 25		0.00572965116297112	1.00574609700885
vPRA	Over 85		-0.057088618453411	0.944510364609943
vPRA	Over 50		-0.0244475142507819	0.97584890573526
Recipient Match Age			0.0386983276945029	1.03945686099918
Recipient Match Age	Under 70		0.0449788476139089	1.04600573415757
Recipient Match Age	Over 75		-0.115959791542542	0.89051102865244
Donor Age	Under 70		-0.0805429695503833	0.922615258369165
Donor Age			-0.0789767598566645	0.92406139951492
Donor Age	Over 75		-0.011469352460997	0.98859616982331
Zero MM			0.114616545859406	1.12144333302503
MM Broad HLA-A		1	-0.0269362904778526	0.973423255879343
MM Broad HLA-A		2	-0.0013058038560025	0.998695048334881
MM Broad HLA-B		1	0.116155746267968	1.12317078817019
MM Broad HLA-B		2	0.0259413654873447	1.02628077123636
MM Split HLA-DR		1	-0.139287265440917	0.869978077922753
MM Split HLA-DR		2	-0.254029301666557	0.775669073316938
Years on Dialysis			0.0727104636986771	1.0754191189594

Table S4: Number of waiting list registrations per year used as model Input

Year	Waiting list Registrations
2006	3027
2007	3169
2008	3306
2009	3147
2010	3453
2011	3496
2012	3225
2013	2796
2014	2848
2015	2793
2016	3036

Table S5: Software environments and packages used in data processing and simulation

Environment	Package / Library	Version
R	R Core Software (9)	4.5.0
	tidyverse (10)	2.0.0
	reticulate (11)	1.42.0
	survival (12,13)	3.8-3
	ggsurvfit	1.1.0
	gt (15)	1.0.0
	gtsummary (16)	2.1.0
	cowplot (17)	1.1.3
	here (18)	1.0.1
	janitor (19)	2.2.1
Python	Python	3.11
	Mesa (20)	2.2.4
	NumPy (21)	1.26.3
	pandas (22)	2.1.4
	SciPy (23)	1.11.4
	lifelines (24)	0.28.0
	matplotlib (25)	3.8.0
	seaborn (26)	0.13.0

Table S6: Comparison of waiting times between model and national registry data

For the model, the median (IQR) is calculated across all pooled simulations.

**Characteristic**	**Overall**[1]	Age group (years)		
		**< 18**[1]	**>=65**[1]	**18 - 65**[1]
**Transplant Registry**				
List to Transplant Time (years)	3.40 (1.13, 6.19)	0.90 (0.40, 1.76)	1.57 (0.51, 3.51)	4.79 (2.01, 6.99)
Dialysis to Transplant Time (years)	5.81 (3.27, 7.91)	1.81 (1.03, 2.67)	3.85 (2.37, 5.47)	6.88 (4.64, 8.57)
Unknown	235	91	40	104
**Model**				
List to Transplant Time (years)	4.55 (1.55, 7.11)	0.41 (0.17, 0.90)	2.08 (0.76, 4.43)	5.94 (3.29, 7.73)
Dialysis to Transplant Time (years)	6.95 (4.12, 8.73)	1.15 (0.61, 1.84)	4.33 (2.67, 6.63)	7.86 (6.20, 9.20)

Table S7: Scenario analysis of omitting the ESP

Comparison of a scenario with only ETKAS rules used for allocation ("ESP omitted") and allocation rules based on the Eurotransplant allocation algorithm currently in place ("no changes"). Per scenario, data from five model runs are aggregated. Donor and recipient ages are presented both as a continuous variable and grouped into predefined age groups.

For continuous variables, the median (IQR) is calculated across all pooled simulations. For categorical variables, the percentage is first calculated within each model run, and then the median [min./max.] across model runs is reported.

Abbreviations: HLA = Human Leukocyte Antigen; vPRA = virtual Panel Reactive Antibodies

**Characteristic**	**No Changes**<br>N = 97,584	**ESP Omitted**<br>N = 97,583
Age Gap	-1 (-10, 8)	-3 (-16, 10)
Age Donor	56 (45, 67)	56 (45, 67)
Age Recipient	56 (46, 65)	53 (44, 62)
Recipient Age Group		
<18	4.8% [4.6%–4.9%]	4.8% [4.5%–5.1%]
18-64	66.6% [66.2%–66.7%]	79.1% [78.8%–79.2%]
>=65	28.7% [28.4%–29.0%]	16.1% [16.0%–16.5%]
Donor Age Group		
<18	3.3% [3.1%–3.4%]	3.4% [3.3%–3.4%]
18-64	68.3% [67.8%–69.0%]	68.1% [67.8%–68.2%]
>=65	28.5% [27.7%–28.8%]	28.6% [28.4%–28.8%]
Recipient Blood Group		
A	44.0% [43.7%–44.2%]	44.4% [43.7%–44.5%]
AB	5.0% [4.6%–5.1%]	4.9% [4.8%–4.9%]
B	11.3% [11.2%–11.4%]	11.3% [11.1%–11.4%]
O	39.9% [39.4%–40.1%]	39.5% [39.3%–40.3%]
Donor Blood Group		
A	44.0% [43.7%–44.2%]	44.4% [43.7%–44.5%]
AB	5.0% [4.6%–5.1%]	4.9% [4.8%–4.9%]
B	11.3% [11.2%–11.4%]	11.3% [11.1%–11.4%]
O	39.9% [39.4%–40.1%]	39.5% [39.3%–40.3%]
vPRA		
0%	85.1% [85.0%–85.2%]	83.7% [83.3%–83.9%]
>0%-<85%	11.9% [11.8%–12.0%]	12.6% [12.4%–12.8%]
>=85%	3.0% [2.9%–3.1%]	3.7% [3.7%–4.0%]
HLA ABDR Mismatches		
0	15.4% [15.0%–15.8%]	19.4% [19.1%–19.8%]
1	6.0% [5.8%–6.1%]	7.8% [7.7%–8.1%]
2	22.1% [21.8%–22.5%]	27.5% [26.8%–28.1%]
3	27.4% [26.9%–28.0%]	31.5% [31.3%–31.7%]
4	15.6% [15.4%–16.4%]	11.2% [11.1%–11.8%]
5	9.5% [9.2%–9.6%]	2.2% [2.0%–2.3%]
6	3.8% [3.7%–3.8%]	0.2% [0.2%–0.3%]
Recipient Region		
Baden-Württemberg	11.1% [10.9%–11.3%]	11.1% [10.9%–11.5%]
Bayern	15.1% [15.0%–15.5%]	14.9% [14.6%–15.1%]
Mitte	12.2% [12.0%–12.3%]	12.2% [11.9%–12.5%]
Nord	16.7% [16.5%–16.8%]	16.6% [16.3%–16.8%]
Nord-Ost	11.2% [11.0%–11.4%]	11.3% [11.2%–11.6%]
Nordrhein-Westfalen	22.0% [21.7%–22.1%]	22.0% [21.8%–22.6%]
Ost	11.6% [11.5%–11.9%]	11.7% [11.5%–12.0%]
Donor Region		
Baden-Württemberg	11.4% [11.1%–11.5%]	11.2% [11.1%–11.7%]
Bayern	15.5% [15.4%–15.9%]	15.2% [15.0%–15.8%]
Mitte	11.9% [11.6%–12.2%]	11.7% [11.5%–12.1%]

Characteristic	No Changes<br>N = 97,584	ESP Omitted<br>N = 97,583
Nord	16.7% [16.5%–16.9%]	16.5% [16.1%–16.9%]
Nord-Ost	11.3% [11.1%–11.4%]	11.4% [11.2%–11.5%]
Nordrhein-Westfalen	20.9% [20.6%–21.1%]	21.2% [20.8%–21.4%]
Ost	12.3% [12.2%–12.6%]	12.5% [12.3%–12.9%]
Donor Location Relative to Recipient		
Home country	14.4% [14.4%–15.0%]	18.2% [17.9%–18.5%]
Regional	85.6% [85.0%–85.6%]	81.8% [81.5%–82.1%]

Table S8: Frequency of regions used as model input for candidates

Region	Percentage
Baden-Württemberg	10.9067516594052%
Bayern	14.5150442995409%
Mitte	12.7810754700430%
Nord	16.4829380976052%
Nordost	11.4243106523583%
Nordrhein-Westfalen	23.2258253165297%
Ost	10.6640545045177%

Table S9: Frequency of blood groups used as model input for candidates

Blood Group	Percentage
A	42.6359527627934%
AB	5.6451377751859%
B	12.7627934101181%
O	38.9561160519026%

Table S10: Frequency of regions used as model input for organs

Region	Percentage
Baden-Württemberg	11.2878520089549%
Bayern	15.4353717450218%
Mitte	11.8357487922705%
Nord	16.5134912218687%
Nordost	11.3055260987392%
Nordrhein-Westfalen	21.2206904677742%
Ost	12.4013196653706%

Table S11: Frequency of blood groups used as model input for organs

Blood Group	Percentage
A	44.0269966254218%
AB	4.8931383577053%
B	11.2710911136108%
O	39.8087739032621%

Supplemental Figures

Figure S1: Flowchart of the discrete-time simulation model for organ allocation and transplantation

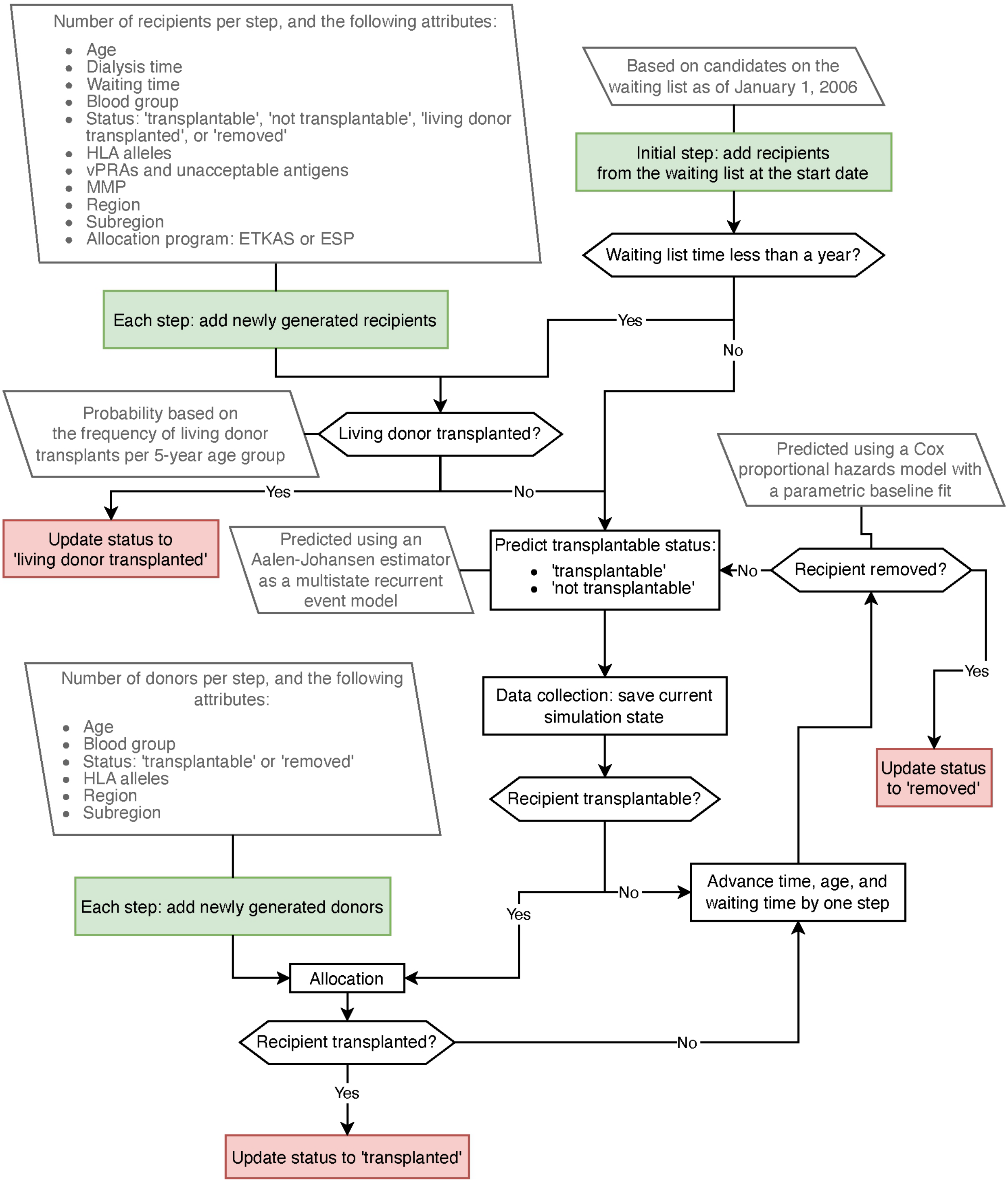

Abbreviations: ESP = European Senior Program; ETKAS = Eurotransplant Kidney Allocation System; HLA = Human Leukocyte Antigen, vPRAs = virtual Panel Reactive Antibodies

Figure S2: Input parameters for the simulated allocation model

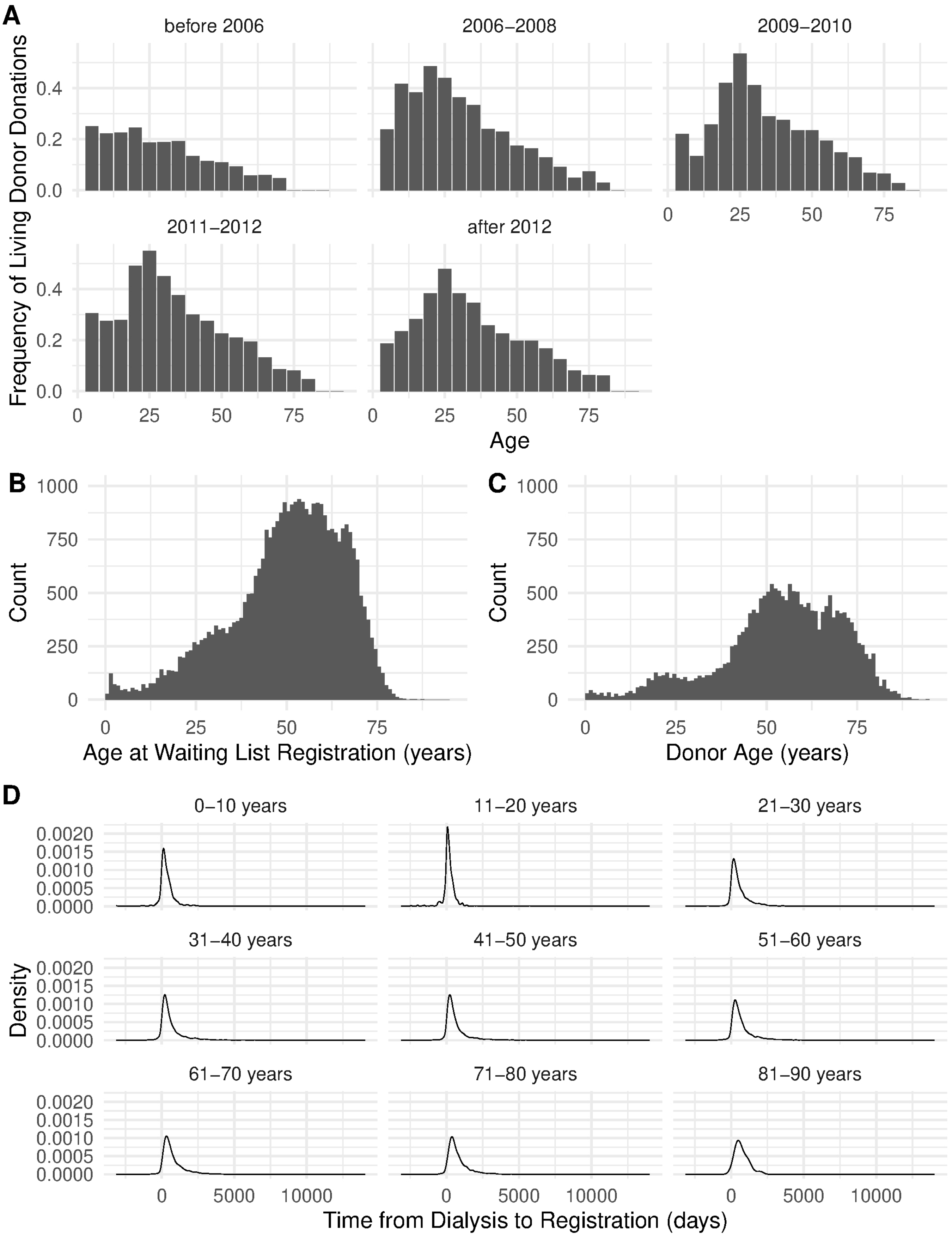

The discrete-time simulation model relied on different input parameters. The parameters were derived from the German national transplant registry.

(A) Frequency of living donor kidney transplants, among new registrations on the waiting list, stratified by registration year and grouped into five-year age band.
(B) Age distribution of candidates on the waiting list at registration. Used for resampling of candidates age in the model. Shown is the count per age (bin width = 1 year).
(C) Donor age distribution used for resampling of organ donor age in the model. Shown is the count of donors per age (bin width = 1 year).
(D) Kernel density estimation of the time (in days) from dialysis initiation to waiting list registration, stratified by candidate age at registration as indicated.

Figure S3: Estimation of transplantability status used inside the simulation model

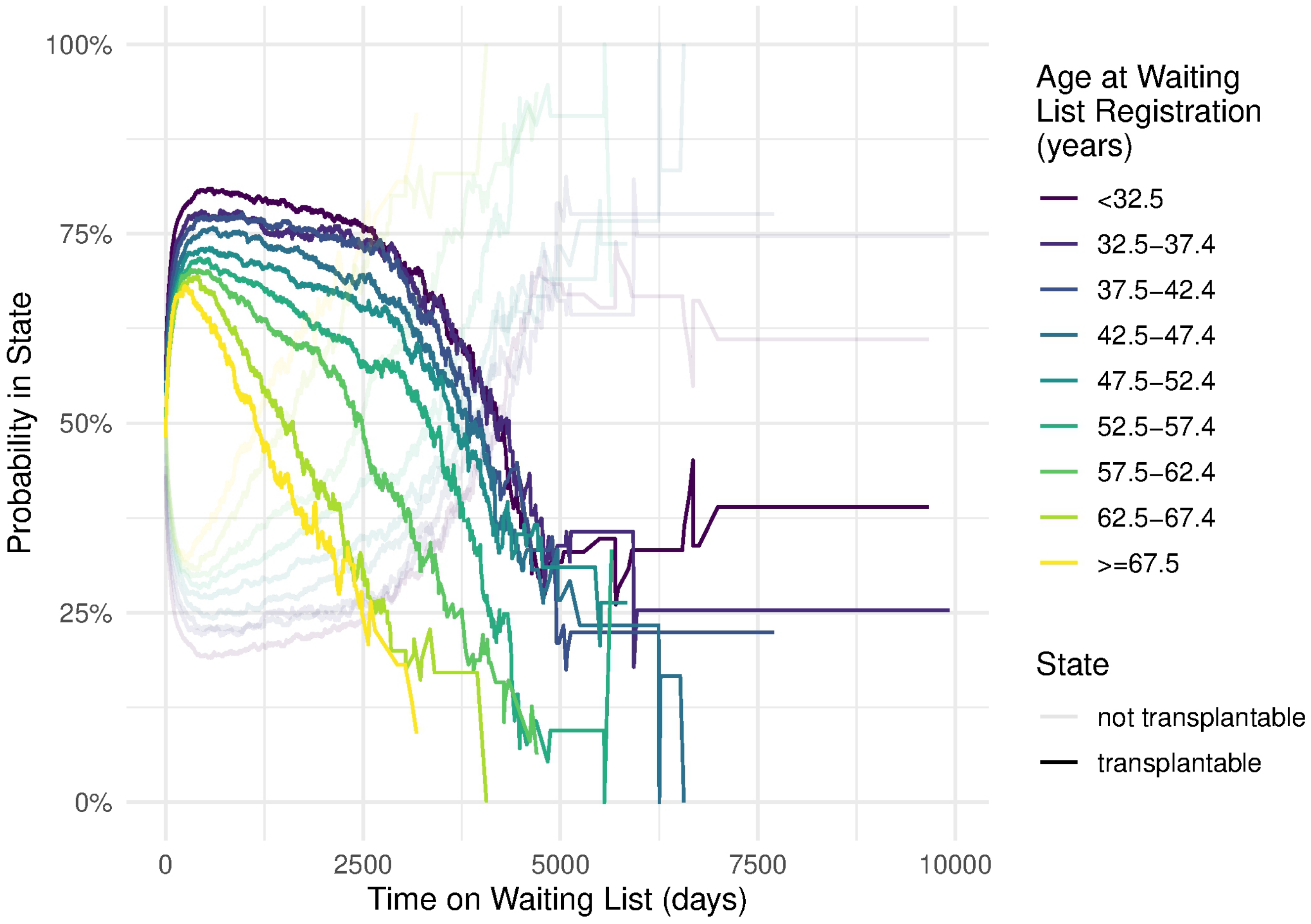

For prediction of the current transplant status of a candidate, an Aalen-Johansen estimator was fitted within a multistate recurrent event framework. The model was fitted to data from the German national transplant registry and stratified by age at waiting list registration, as indicated.

Figure S4: Flowchart of organ allocation in the model

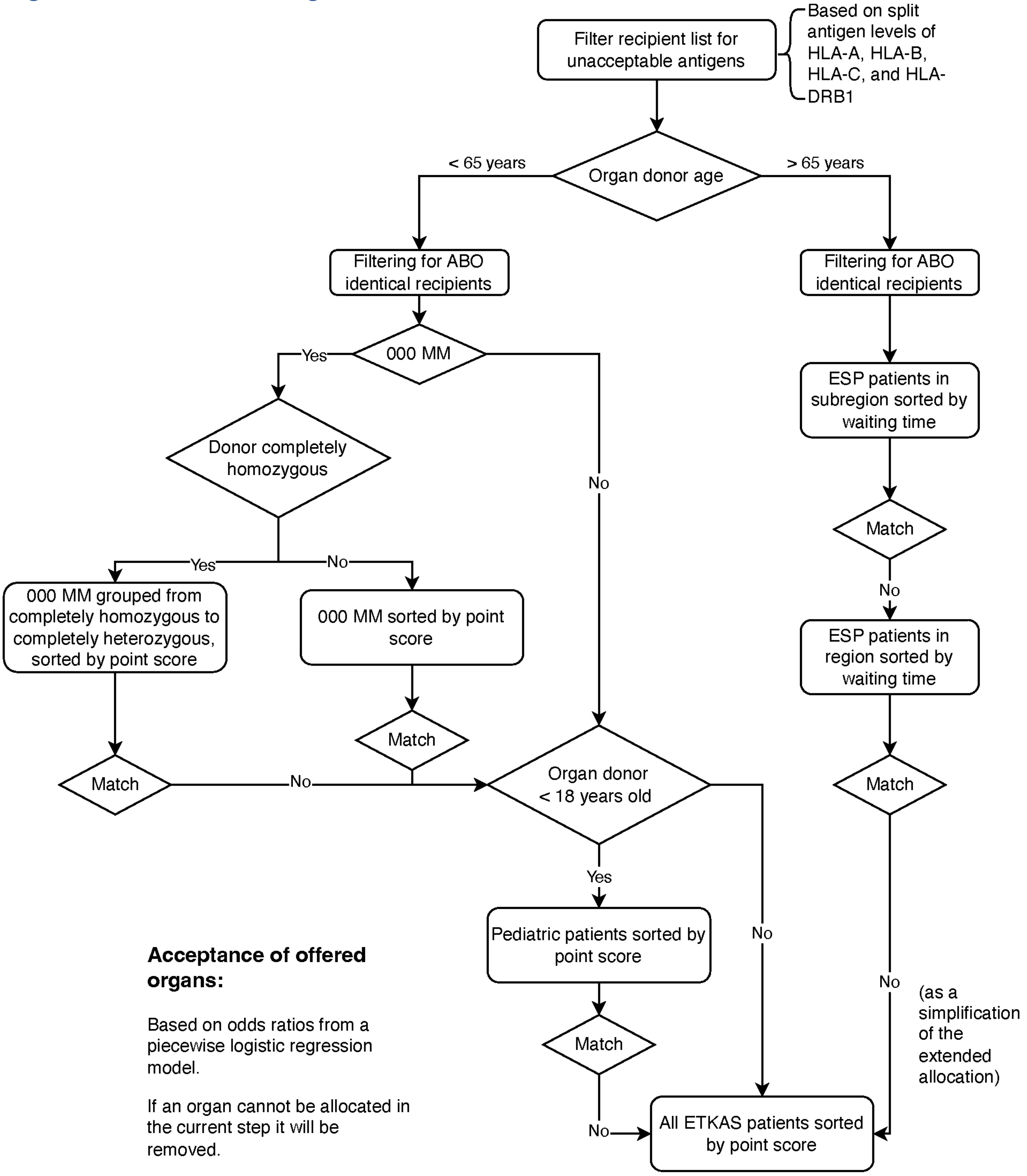

Acceptance of offered organs:

Based on odds ratios from a piecewise logistic regression model.

If an organ cannot be allocated in the current step it will be removed.

Abbreviations: 000 MM = Zero Mismatch; ESP = European Senior Program; ETKAS = Eurotransplant Kidney Allocation System; HLA = Human Leukocyte Antigen, vPRAs = virtual Panel Reactive Antibodies

Figure S5: Estimation of removal from the waiting list within the simulation model.

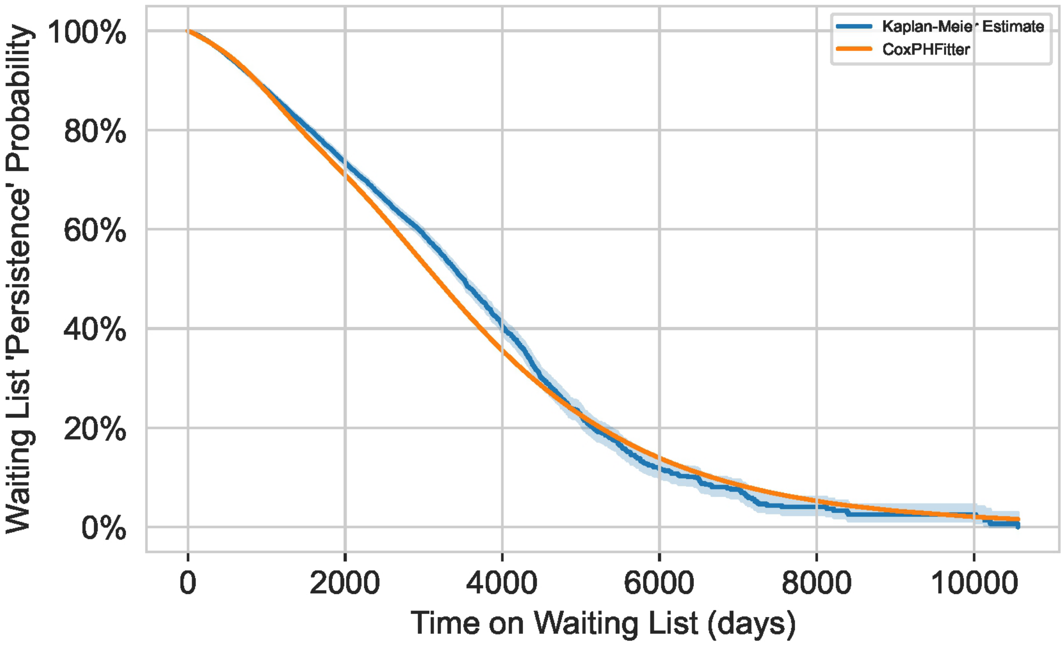

Kaplan-Meier Estimate						
At risk	31517	9794	858	81	12	5
Censored	0	26201	34953	35440	35479	35484
Events	0	5051	7141	7511	7549	7553

To predict removal from the waiting list, a Cox proportional hazards model with a parametric baseline hazard (estimated using cubic splines) was used. The square root of the time from dialysis initiation to waiting list registration was included as a covariate. Candidates were stratified by age at waiting list registration into the following groups: 0–29, 30–39, 40–44, 45–49, 50–54, 55–59, 60–64, 65–69, 70–74, 75–79, and 80 years and older. The Input data were left censored for registrations before January 1, 2006, and right censored at time of transplantation or January 1, 2017.

The plot shows Kaplan-Meier estimates and the corresponding Cox model curve, pooled across all age groups, based on data from the German national transplant registry.

Figure S6: Comparison of waiting list removals between model data and data from the

A

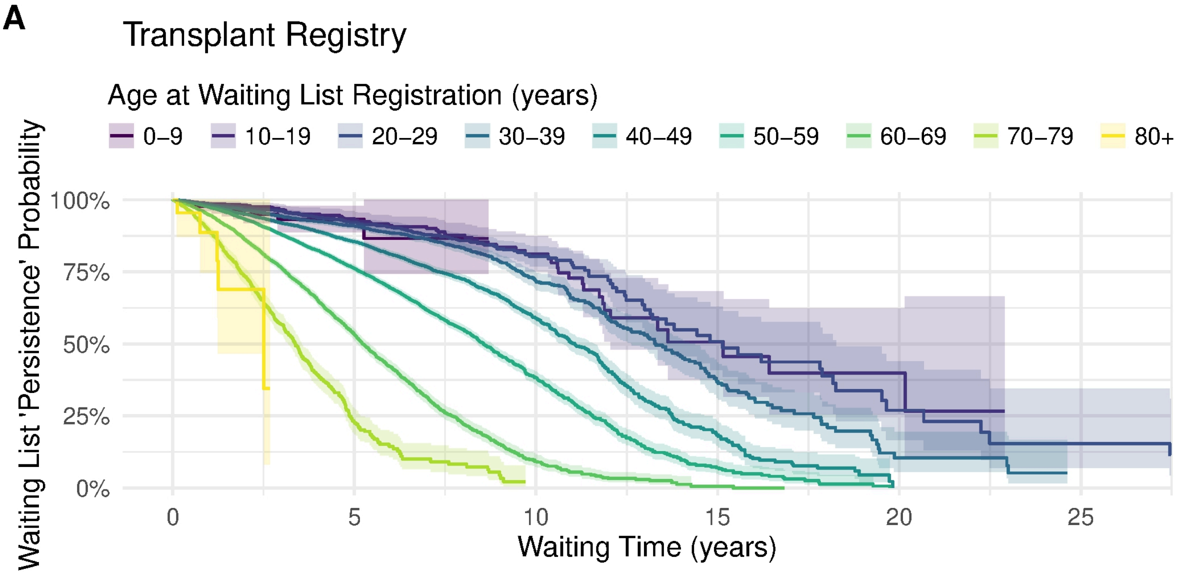

At Risk (Events)

549 (0)	15 (13)	0 (14)	0 (14)	0 (14)	0 (14)
1030 (0)	221 (26)	63 (44)	10 (57)	4 (59)	0 (60)
2552 (0)	890 (102)	150 (163)	24 (185)	7 (194)	5 (197)
3711 (0)	1849 (222)	315 (417)	62 (499)	6 (525)	1 (527)
7326 (0)	3398 (728)	440 (1274)	38 (1434)	0 (1459)	0 (1459)
9078 (0)	3610 (1427)	371 (2411)	21 (2595)	0 (2610)	0 (2610)
7733 (0)	1013 (1724)	47 (2209)	1 (2240)	0 (2242)	0 (2242)
2239 (0)	60 (566)	0 (596)	0 (596)	0 (596)	0 (596)
24 (0)	0 (5)	0 (5)	0 (5)	0 (5)	0 (5)

B

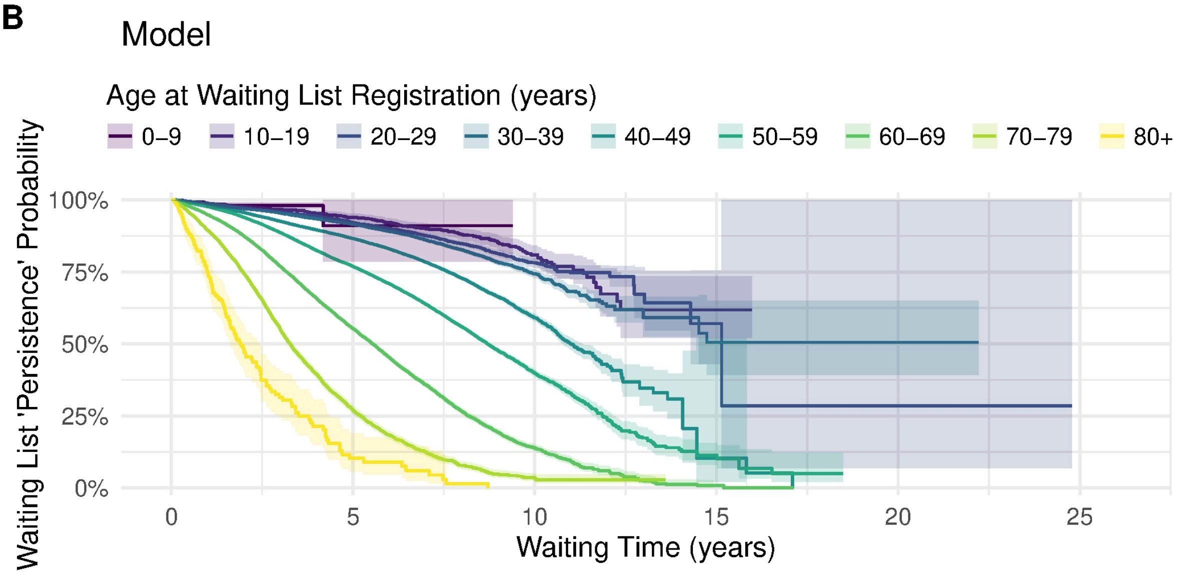

At Risk (Events)

2791 (0)	2 (16)	0 (16)	0 (16)	0 (16)	0 (16)
5406 (0)	1067 (84)	170 (168)	1 (183)	0 (183)	0 (183)
12725 (0)	4994 (503)	499 (898)	2 (917)	5 (918)	0 (918)
18819 (0)	10172 (1035)	778 (1998)	11 (2051)	5 (2051)	0 (2051)
36844 (0)	18407 (3356)	1115 (6059)	4 (6196)	0 (6198)	0 (6198)
45673 (0)	20266 (7306)	818 (11919)	10 (12089)	0 (12093)	0 (12093)
38212 (0)	6121 (8947)	203 (11330)	1 (11412)	0 (11414)	0 (11414)
12025 (0)	698 (3709)	9 (4090)	0 (4091)	0 (4091)	0 (4091)
259 (0)	8 (134)	0 (141)	0 (141)	0 (141)	0 (141)

German national transplant registry

(A) Kaplan-Meier curves fitted to registry data.
(B) Kaplan-Meier curves fitted to pooled data from the five runs of the model.
Kaplan-Meier curves for waiting list persistence probability are shown. Age at waiting list registration was stratified as indicated. Left censoring was applied for candidates who entered the waiting list prior to January 1, 2006. Candidates were right censored at time of transplantation. Candidates neither transplanted nor removed from the waiting list until January 1, 2017, were censored at that date.

Figure S7: Validation of model reliability trough comparison with historical data from the German national transplant registry

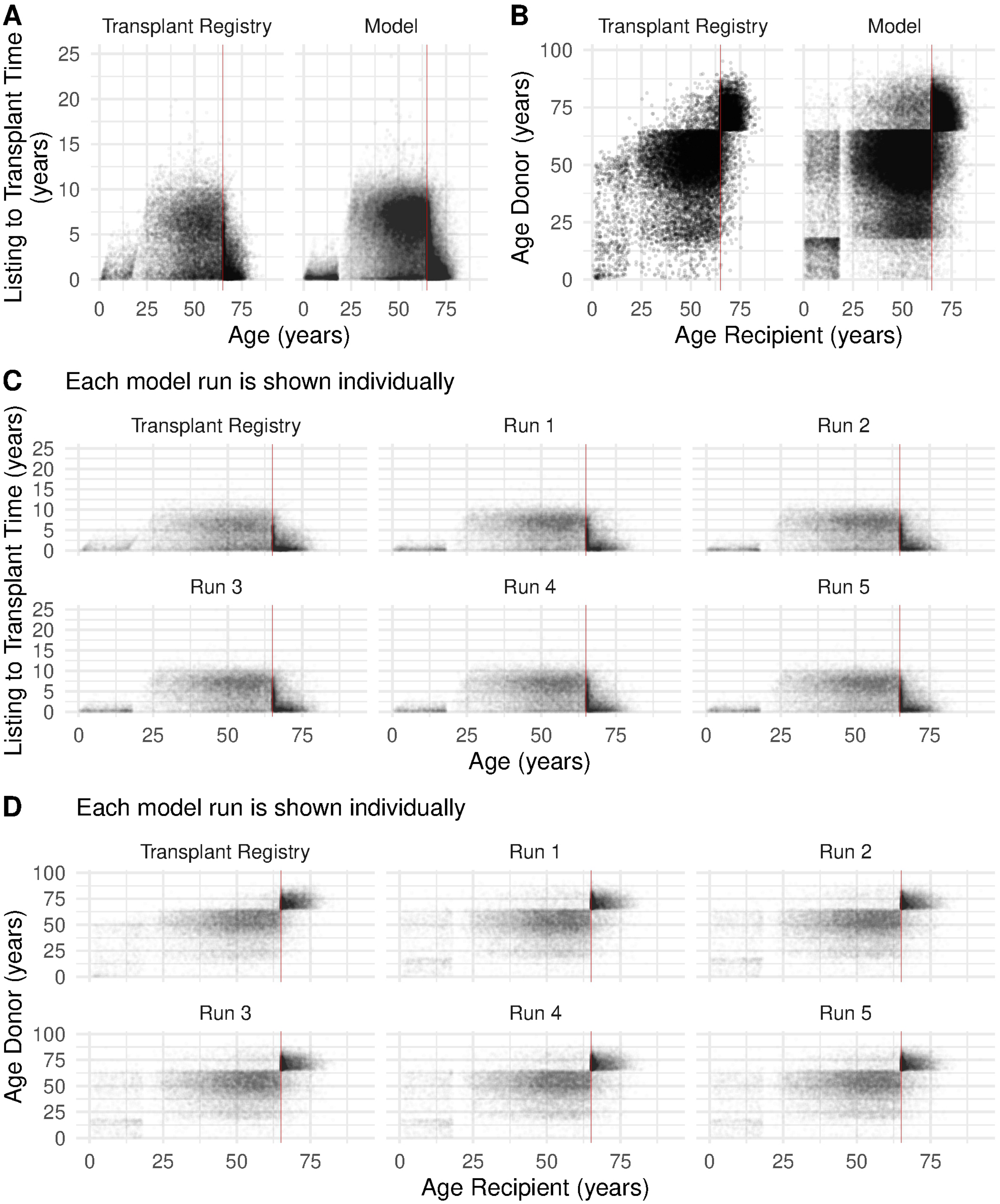

(A) Time from waiting list registration to transplantation (in years) is plotted against recipient age at transplantation. Each dot represents one transplant. For the discrete-time simulation model, data were pooled from five independent model runs.

(B) Recipient age is plotted against donor age at the time of transplantation. Each dot represents one transplant. For the discrete-time simulation model, data were pooled from five independent model runs.
(C) Time from waiting list registration to transplantation (in years) is plotted against recipient age at transplantation. Each dot represents one transplant. Data are displayed separately for each of the five model runs.
(D) Recipient age is plotted against donor age at the time of transplantation. Each dot represents one transplant. Data are displayed separately for each of the five model runs.

Figure S8: Post transplant model behavior

A

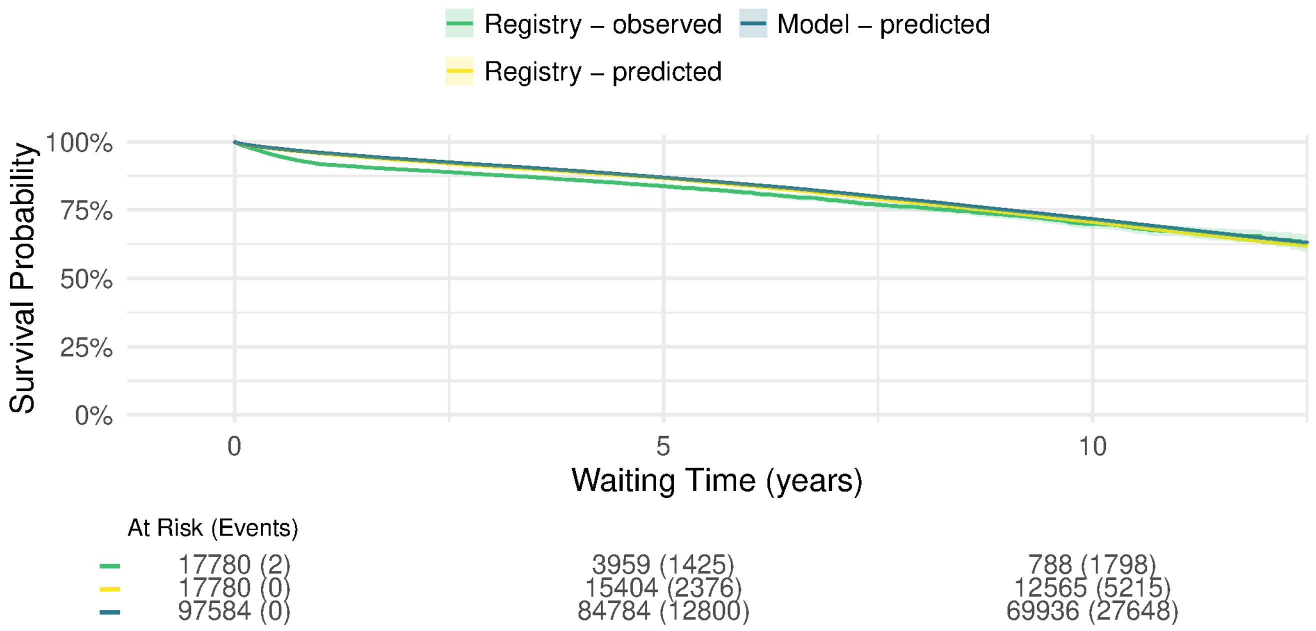

B

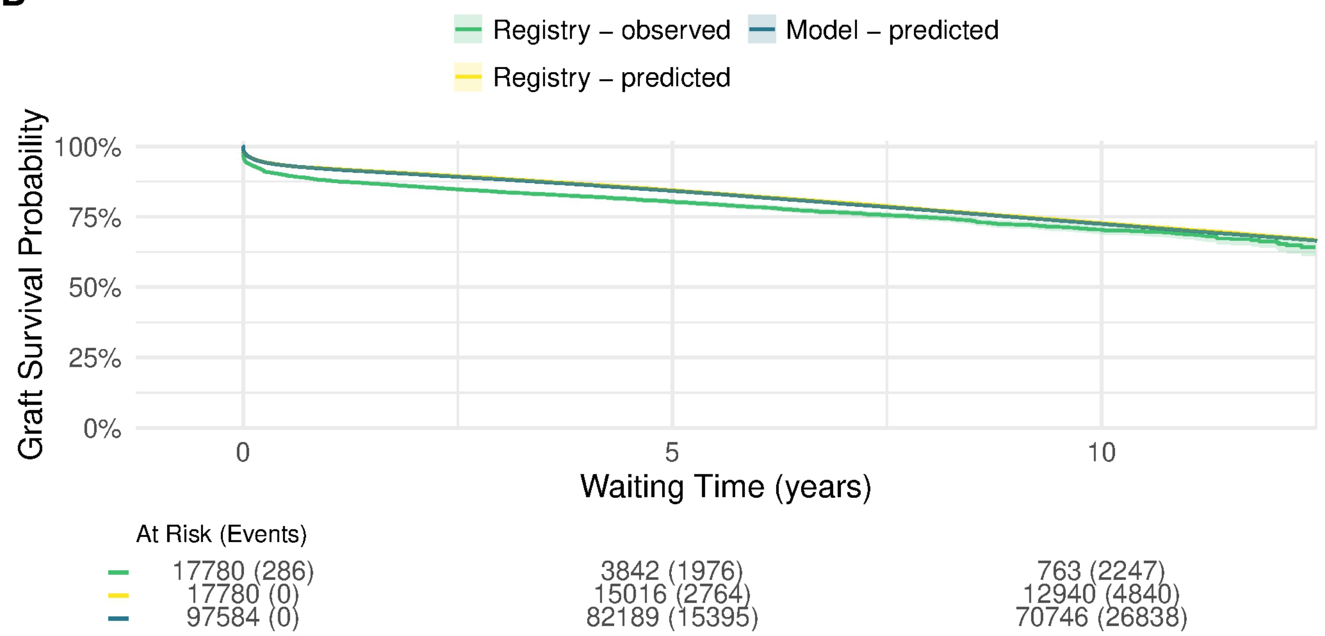

(A) Patient survival probability is displayed. The observed patient survival is estimated from registry data using the Kaplan-Meier estimator.

(B) Graft survival probability is displayed. The observed graft survival is estimated from registry data using the Aalen-Johansen estimator, which accounts for competing risks.

Predicted survival was simulated in both graphs using a Royston-Parmar model published by Coemans et al, once using data from the transplant registry and once using the pooled data from the five runs of the model.

Figure S9: Comparison of waiting times and age gaps between both scenarios

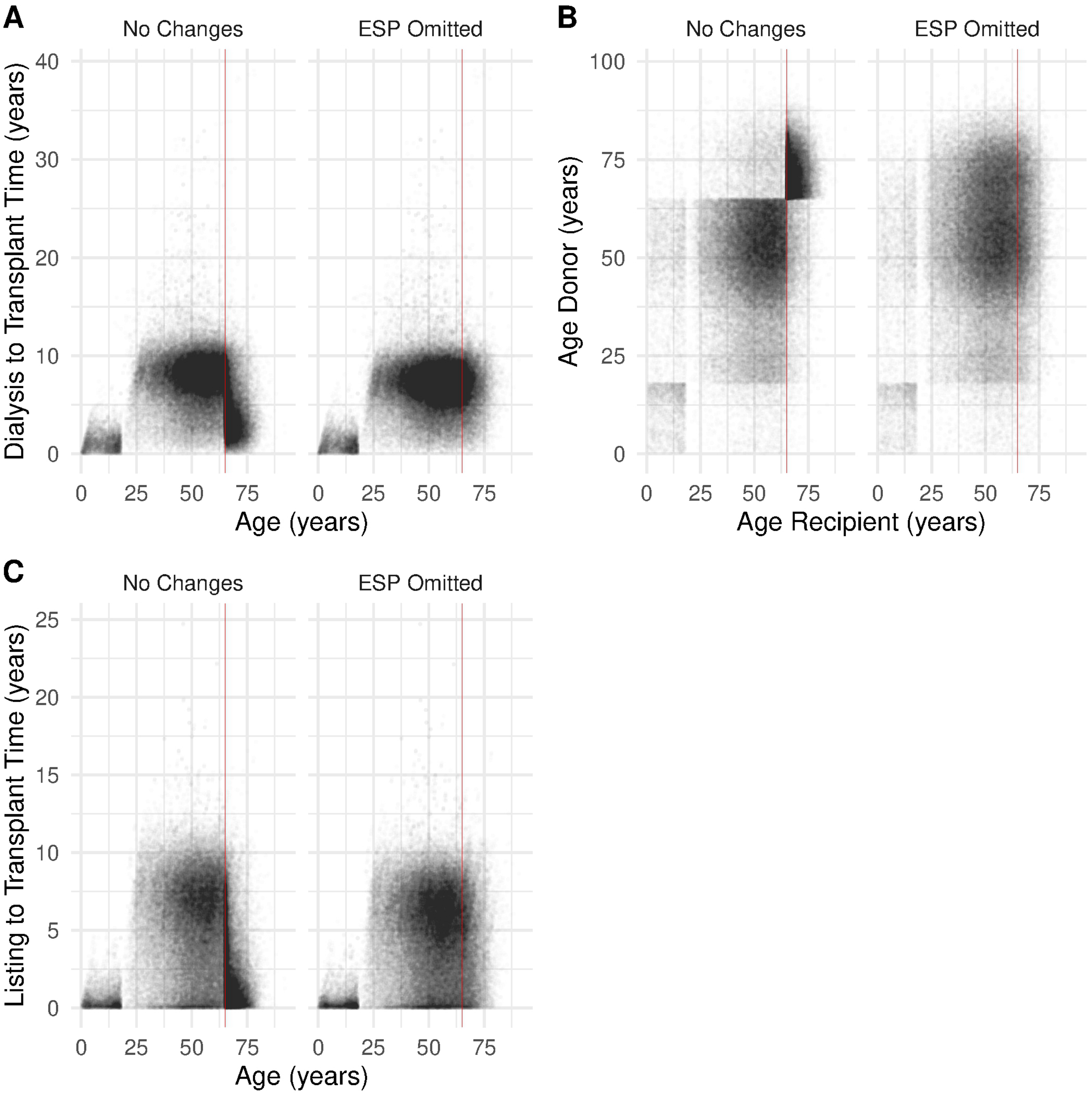

Two different scenarios were compared; simulation of the Eurotransplant allocation rules, as currently in place (“no changes”), and simulation of a scenario where only the ETKAS rules are used for allocation (“ESP omitted”). Data were pooled from five independent simulation runs per scenario. Each dot represents one transplantation.

(A) Dialysis to transplant time in years for all transplanted patients is plotted against age at transplantation in years.

(B) Recipient age is plotted against donor age at the time of transplantation.

(C) Time from waiting list registration to transplantation (in years) is plotted against recipient age at transplantation.

Abbreviations: ESP = European Senior Program; ETKAS = Eurotransplant Kidney Allocation System

Figure S10: Analysis of post-transplant outcome under both allocation scenarios

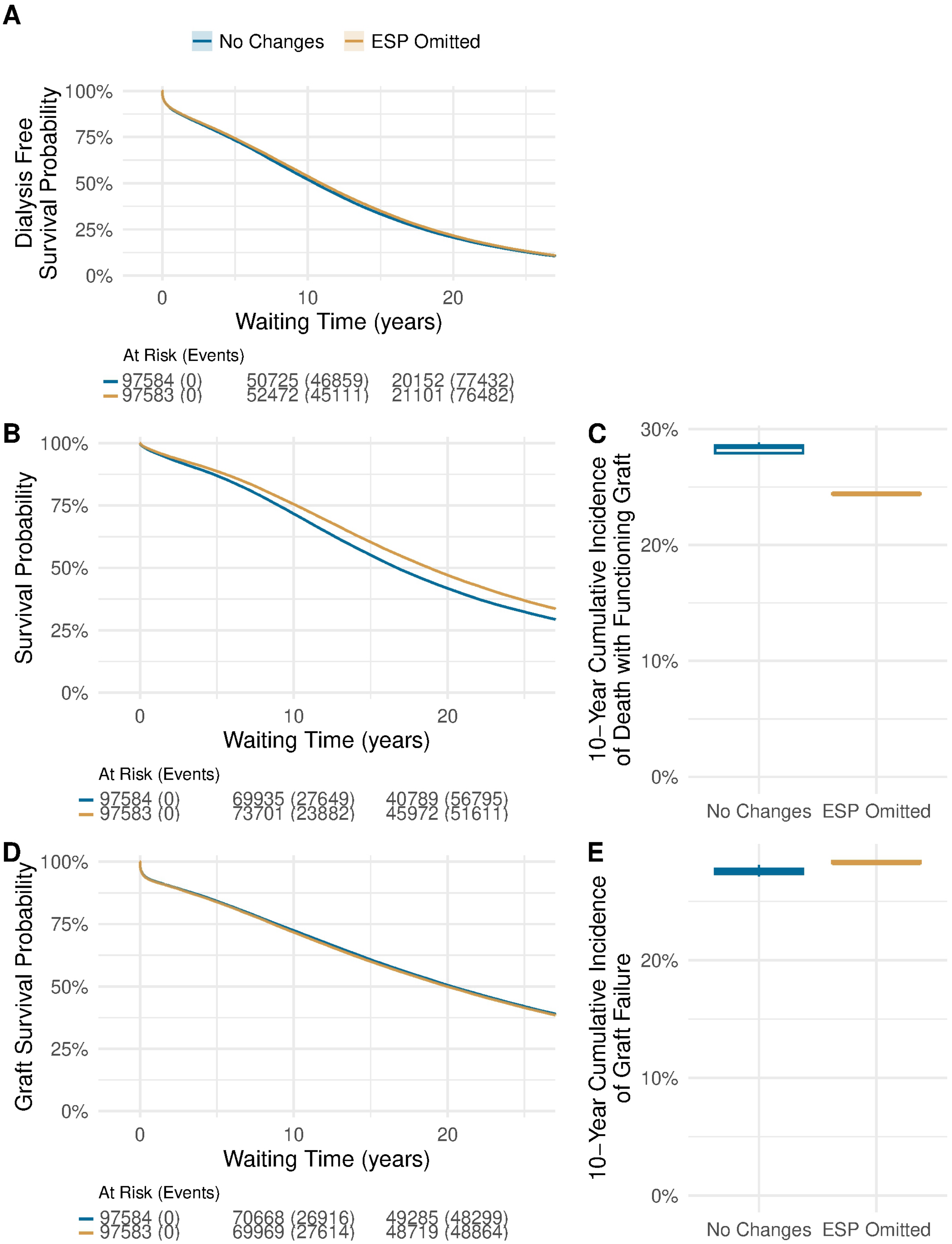

Predicted survival was simulated using a Royston-Parmar model. The data was pooled for each scenario from five model runs. Comparison took place between simulation of a scenario with the Eurotransplant allocation rules currently in place („no changes“) and a scenario in which only the ETKAS rules were applied, omitting ESP (“ESP omitted”).
Cumulative incidence was estimated for each simulation run using a competing risks Aalen-Johansen estimator, with median and interquartile ranges reported across runs.
(A) Dialysis free survival probability is displayed.
(B) Survival probability is displayed.
(C) 10-year cumulative incidence of death with functioning graft is displayed.
(D) Graft survival probability is displayed.
(E) 10-year cumulative incidence of graft failure is displayed.
